# Identification of low-dose multidrug combinations for sunitinib-naive and pre-treated renal cell carcinoma

**DOI:** 10.1038/s41416-020-0890-y

**Published:** 2020-05-22

**Authors:** Magdalena Rausch, Andrea Weiss, Joanna Achkhanian, Andrei Rotari, Patrycja Nowak-Sliwinska

**Affiliations:** 1 Molecular Pharmacology Group, Institute of Pharmaceutical Sciences of Western Switzerland, 1 Rue Michel-Servet, 1211 Geneva 4, Switzerland; 2 Translational Research Center in Oncohaematology, 1 Rue Michel-Servet, 1211 Geneva 4, Switzerland

**Keywords:** Targeted therapies, Renal cell carcinoma

## Abstract

**Background:**

Combinations of drugs can improve the efficacy of cancer treatment, enable the reduction of side effects and the occurrence of acquired drug resistance.

**Methods:**

We approached this challenge mathematically by using the validated technology called the Therapeutically Guided Multidrug Optimization (TGMO) method. In a set of genetically distinct human renal cell carcinoma (RCC) cell lines, either treated chronically with sunitinib (−ST) or sunitinib-naive, we identified cell line-specific low-dose-optimised drug combinations (ODC).

**Results:**

Six cell-type-specific low-dose drug combinations for three sunitinib-naive as well as three sunitinib pre-treated cells were established. These ODCs effectively inhibited the RCC cell metabolic activity while being ineffective in non-cancerous cells. Based on a single screening test and three searches, starting with ten drugs, we identified highly efficacious drug mixtures containing four drugs. All ODCs contained AZD4547 (FGFR signalling pathway inhibitor) and pictilisib (pan-phosphatidylinositol 3-kinase inhibitor), but varied in the third and fourth drug. ODC treatment significantly decreased cell metabolic activity (up to 70%) and induced apoptosis, independent of the pretreatment with sunitinib. The ODCs outperformed sunitinib, the standard care for RCC. Moreover, short-term starvation potentiated the ODC activity. The translation of the 2D-based results to 3D heterotypic co-culture models revealed significant inhibition of the spheroid growth (up to 95%).

**Conclusion:**

We demonstrate a promising low-dose drug combination development to obtain drug combinations effective in naive as well as resistant tumours. Nevertheless, we emphasise the need for further mechanistic investigation and preclinical development.

## Background

The clinical benefit of using low-dose drug combinations to treat complex diseases has become a major interest in the medical community because of the advantages, such as enhanced efficacy, lack of intrinsic or acquired resistance and the limitation of dose-related toxicities. This particularly holds true in the case of cancer therapy, where in many cases, adequate treatments are still lacking due to intra-patient and/or inter-/intra-tumour heterogeneity. These issues together restrict the efficacy and overall success of many treatment strategies that are currently used for the clinical management of cancer.^1,2^ The use of targeted anticancer agents and genotype-based treatments has provided attractive avenues for the improvement of therapy, and has shown important clinical successes.^3,4^ For the treatment of renal cell carcinoma (RCC), targeted agents have generally shown good efficacy. Sunitinib (Sutent^TM^) is one of the first-line treatment therapies for RCC.^5^ Many preclinical and clinical efforts for this indication have focused on identifying effective combinations of existing targeted agents using either simultaneous or sequential administration to maximise their clinical benefits. Large studies, such as the BEST, RECORD, INTORACT, CALGB, TORAVA, CheckMate 214 and Keynote-426 studies,^6–11^ investigated first-line combination regimens and, in most cases, did not show superior outcomes over single agents.^12^ Instead of enhanced efficacy, substantial toxicity has been a recurrent observation in these studies,^12^ even when combinations are designed to target complementary pathways. Therefore, the application of appropriate methods to design optimal drug combination regimens with optimal drug selections and use at appropriate doses is of major importance.

Properly designed drug combinations may help to overcome some of the current limitations of cancer therapy by challenging the robust biological processes.^13,14^ The limited success of clinically tested drug combinations thus far can largely be attributed to intolerable toxicities, lack of efficacy and/or acquired drug resistance.^15,16^ Two of the main challenges in defining optimal drug combinations are patient heterogeneity and the broad number of possibilities, where one must consider the candidate drug selection together with their relative dose ratios. These will affect potential synergism or antagonism, as well as toxicity profiles of the drug combinations.

In order to solve these problems, we have developed and applied our technology called streamlined-feedback system control (s-FSC), a strategy for rapid in vitro identification of optimised, synergistic low-dose multidrug combinations.^13,14^ In contrast to other approaches based on pharmacogenetics or high-throughput screening, this phenotypically driven approach based on the statistical design of experiment (DoE) identifies synergistic drug mixtures with minimal experimental effort. Moreover, it does not require background and mechanistic information about the system prior to the start of optimisation.^13,14,16^ Drug combinations are tested in only three iterative searches, and the in vitro data are used to perform an analysis in ‘search rounds’. Each search produces a second-order linear regression model and specified regression coefficients defining these models. This technique helps to discriminate synergistic, additive and antagonistic drug interactions.^14,17^ We have previously applied the s-FSC platform to navigate in the experimental parametric space of ten small-molecule drugs^13,14,17,18^ applied at low doses. In all cases, the rapid, iterative approach of in vitro testing for cell metabolic activity, together with a second-order regression modelling, allowed for the identification of an optimal, synergistic low-dose drug combination.

In this study, we investigated the improvement of RCC treatment via the identification of synergistic drug combinations effective in sunitinib-naive and sunitinib pre-treated RCC cells. We used a new generation of the s-FSC method called the Therapeutically Guided Multidrug Optimization (TGMO), where drug combinations are tested simultaneously on both cancer (in this case RCC cells) and non-cancerous cells (e.g. non-malignant renal cells) at low doses (i.e. below maximal plasma doses, MPD, in humans). This strategy allows the identification of optimised multidrug combinations (ODC) acting with high efficacy and selectivity.^19^ Starting with a set of ten small-molecule-based compounds, we have identified four-drug combinations applied simultaneously specific for each cell line. The individually optimised regimen revealed different drug-dose parameters for each cell type. The drug mixture optimised for A489 sunitinib pre-treated (A489-ST) cells contained AZD4547 (an inhibitor of the fibroblast growth factor receptor (FGFR) tyrosine kinase family),^20^ AZD8055 (an ATP-competitive mammalian target of rapamycin kinase (mTORC1) inhibitor),^21^ pictilisib (GDC-0941, a pan-phosphatidylinositol 3-kinase inhibitor),^22^ and selumetinib (an ATP-independent inhibitor of mitogen-activated protein kinase (MEK or MAPK/ERK kinase) 1 and 2).^23^ This drug combination was effective at inhibition of cell metabolic activity in both 2D and heterotypic 3D co-cultures, and induced G1 arrest, p21 expression as well as apoptosis.

## Methods

### Cell culture

Human clear-cell RCC cell lines A498, Caki-1 and 786-O were purchased at ATCC. All three cell lines are of epithelial origin, with A498 being isolated from a primary clear-cell RCC lesion, Caki-1, from a skin metastasis, and 786-O from a primary clear-cell adenocarcinoma.

Human embryonic non-cancerous HEK-293T cells were purchased in ATCC and generously donated by Dr. Dormond (CHUV, Lausanne, Switzerland) and human immortalised endothelial cells’ ECRF24 by Prof. AW Griffioen (Angiogenesis Laboratory, UMC Amsterdam). Normal human dermal fibroblast adult (NHDFα) cells were purchased from Vitaris (Baar, Zug, Switzerland). 786-O and HEK-293T cells were cultured at 37 °C in RPMI medium, A498 and Caki-1 cells in DMEM medium and ECRF24 in 50:50 RPMI:DMEM medium, supplemented with 10% foetal bovine serum (Biowest, Nuaillé, France, S1810-500) and 1% penicillin/streptomycin (Bioconcept, Basel, Switzerland, 4-01F00-H). Cells were tested for mycoplasma contamination frequently and authenticated by Microsynth AG (Balgach, St. Gallen, Switzerland). A subset of A498, Caki-1 and 786-O RCC cells was split and chronically treated with 1 µM sunitinib to obtain sunitinib-naive and sunitinib pre-treated cells, referred to as –ST. The cells were screened for the presence of mycoplasma. Cell line identity was confirmed using STR systems from Promega (Zurich, Switzerland) and the following database comparison.

### Small-molecule drugs

All compounds (Supplementary Table S1) were dissolved at the given concentrations in sterile DMSO (Sigma-Aldrich, D8418-50ML). Alisertib (10 mg/mL), axitinib (20 mg/mL), AZD4547 (10 mg/mL), AZD8055 (5 mg/mL), crenolanib (10 mg/mL), Pictilisib (30 mg/mL), icaritin (5 mg/mL), osimertinib (20 mg/mL), saracatinib (20 mg/mL) and selumetinib (20 mg/mL) were purchased from LC Labs (Woburn, MA, USA) or from Selleck Chemicals (Houston Texas, USA). Sunitinib (15 mg/mL) was purchased from Pfizer, and cisplatin (0.5 mg/mL) was purchased from Selleck Chemicals (Houston, Texas, USA). Aliquots were stored at −80 °C and thawed prior to use. A maximal concentration of 0.05% DMSO was allowed for any of the screened conditions and was used as a control (Ctrl). Sunitinib at a concentration of 1 µM as well as cisplatin at 400 µM have been used as positive controls.

### Combinatorial drug screen and TGMO-based screen

The inhibition of the cell metabolic activity was measured to analyse the activity of each drug combination using CellTiter-Glo solution (Promega, G7572). The therapeutic window was determined by comparing the efficacy of each drug combination between cancerous RCC and non-cancerous cell lines. Second-order stepwise linear regression models using Matlab® were applied to model the results as described by Weiss et al.^14^ Statistical measures—Cook’s distance, regression coefficient, root mean square error and data point correlations—defined the accuracy and reliability of regression models for each iteration, with an ANOVA lack-of-fit test confirming the selection of a relevant model structure. After three sequential search rounds, the compounds alisertib, crenolanib and icaritin were excluded from further experiments based on the regression model elimination. The final dose-optimisation step has led to the selection of cell-type-specific four-drug combinations (Table 1).

**Table 1:** Cell line-specific four-drug low-dose combinations and their efficacy in cell metabolic activity inhibition identified in the TGMO-based search.

Cells		A498	A498-ST	Caki-1	Caki-1-ST	786-O	786-O-ST	ECRF24
Drugs	MPC [µM]	Dose in ODC [µM]						
Axitinib	0.02	–	–	–	–	–	–	0.02
AZD4547	0.4	0.4	0.4	0.4	0.4	0.4	0.4	–
AZD8055	0.03	0.02	0.01	0.03	0.03	–	0.03	–
Osimertinib	0.4	0.4	–	–	0.4	–	–	–
Pictilisib	2.0	1	0.5	0.75	2.0	2.0	2.0	0.5
Saracatinib	0.6	–	–	0.6	–	0.6	0.6	0.6
Selumetinib	0.6	–	0.6	–	–	0.6	–	0.6
Efficacy (% Ctrl)		66.3**	71.6**	70.0**	69.6**	67.8**	70.3**	53.5**
±SD		11	8	8	7	16	9	5

**(*p*-value < 0.01) compared to the monotherapies and the DMSO control.

### F-actin-nucleus double staining

Glass inserts were placed in each well of a 24-well plate before seeding. After experimentation, cells, attached to the glass inserts, were fixed for 10 min in 4% PFA solution, washed twice with PBS and permeated in 0.1% Triton-X solution. Fluorescence double staining was performed by placing the glass slides upside down on a drop of staining solution that was positioned on a table area covered with parafilm. F-actin staining with 1:200 dilution of Alexa Fluor 488- conjugated phalloidin (Invitrogen, Carlsbad, California, United States, A12379) was performed for 20 min at RT following nuclear staining with 1:2500 dilution of Dapi for approximately 5 min. Images were taken with a Biotek Citation 3 (BioTek instruments) with corresponding software at the default settings. Pictures were analysed with CellProfiler^TM^ and Adobe® Photoshop.

### Cell cycle and cell fate analysis

Induction of necrosis after combination treatment was analysed using the RealTime-Glo™ Annexin V Apoptosis and Necrosis Assay Kit (Promega, JA1011) following the manufacturer’s instructions. Discrimination of the cell cycle distribution has been performed staining a cell pellet of approximately 5 × 10^5^ cells with 500 µL of FxCycle^TM^ PI/RNase staining solution (Invitrogen, F10797).

### p21 fluorescence imaging

Cells were plated, fixed and permeabilised on glass slides as previously described, and stained with a 1:800 α-p21 antibody (CellSignalling, Leiden, Netherlands, 2947S) overnight at RT in the dark. Glass slides were dipped in PBS several times for washing and afterwards stained with a 1:400 dilution of DyLight488-coupled secondary antibody (ImmunoReagents, Raleigh, NC, USA, Rb-003-V) for 1 h. Glass slides were washed thoroughly and further stained with DAPI as described above.

### Western blot

One-million RCC cells were grown in petri dishes and treated for 2 h either with the Ctrl or the optimised combination treatment. Following two washing steps with ice-cold PBS, cells were lysed in 1× RIPA buffer (Cell Signaling, 9806S) supplemented with phosphatase inhibitor (Roche, Basel, Switzerland, 04906837001) and protease inhibitor (Roche, 11836170001). Protein concentrations of lysates were quantified using Bradford assay (Sigma, Darmstadt, Germany, B6916-500ML). Protein separation of 30 µg of lysate was performed on 4–15% pre-casted polyacrylamide gels (Biorad, Hercules, California, United States, 456-1083). The wet transfer technique was used to blot the proteins to a nitrocellulose membrane. About 5% milk was used to block membranes following incubation with primary (overnight) and fluorochrome-coupled secondary antibodies (30 min); bands from immunoreactive proteins were measured with an Odyssey infrared imaging system (700 nm for α-mouse- and at 800 nm for α-rabbit- stained proteins). We stained for α-tubulin (Cell Signaling, 3873S), MAPK (Cell Signaling, 4695S) and p21 (Cell Signaling, 2947S). Images were obtained with the Licor Odyssey CLx scanner at one default exposure setting. Image Studio^TM^ Lite software was used to quantify the intensity of every single band by adjusting the brightness per blot row for sufficient band quantification.

### Heterotypic 3D co-cultures

Heterotypic RCC 3D co-cultures consisted of 1800 RCC cells co-cultured with 900 NHDFα cells and 300 ECRF24 cells supplemented with 2.5% Matrigel (Corning, Root, Lucerne, Matrigel #354230).^24^ All consumables were pre-cooled, and the mixing of cells and basement membrane was performed on ice to prevent early polymerisation of Matrigel. To promote the spheroid formation, 80 µl/well were seeded in a round-bottom 96-well plate with the cell-repellent surface (Cellstar® 650970), which was centrifuged at 4 ^o^C for 2 min at 1200× rpm. Drug treatment on co-cultured spheroids was performed for 72 h. Growth kinetics and cell metabolic activity (CellTiter-Glo® 3D Cell Viability Assay (Promega, #G9681)) were measured with a Biotek citation 3 with corresponding software at the default settings.

### CellTracker staining

Prior to staining, all cells were washed with PBS. Afterwards, RCC cells were stained for 20 min with 5 µM Green CMFDA, NHDFα for 40 min with 50 µM blue CMAC and ECRF24 cells for 20 min with 10 µM Red CMPTY (C7025 and C34552, Life Technologies) diluted in serum-free medium. Following another washing step with PBS, cells were harvested, and 3D co-cultures established. Spheroid formation was monitored by fluorescent imaging.

### Statistical analysis

The data are presented as the mean of multiple independent experiments. Error bars represent the standard error, unless otherwise specified. Using either one-way ANOVA test with post hoc Tukey’s multiple-comparison test or a student’s *t* test (Graphpad Prism®), significance was determined. Statistically significant values were calculated vs. the control or monotherapeutic regimens, *p* values are specifically indicated in each figure legend and marked with ** or * according to graphs.

## Results

### TGMO-based identification of synergistic four-drug combinations specific to each RCC cell line

**Fig. 1:**
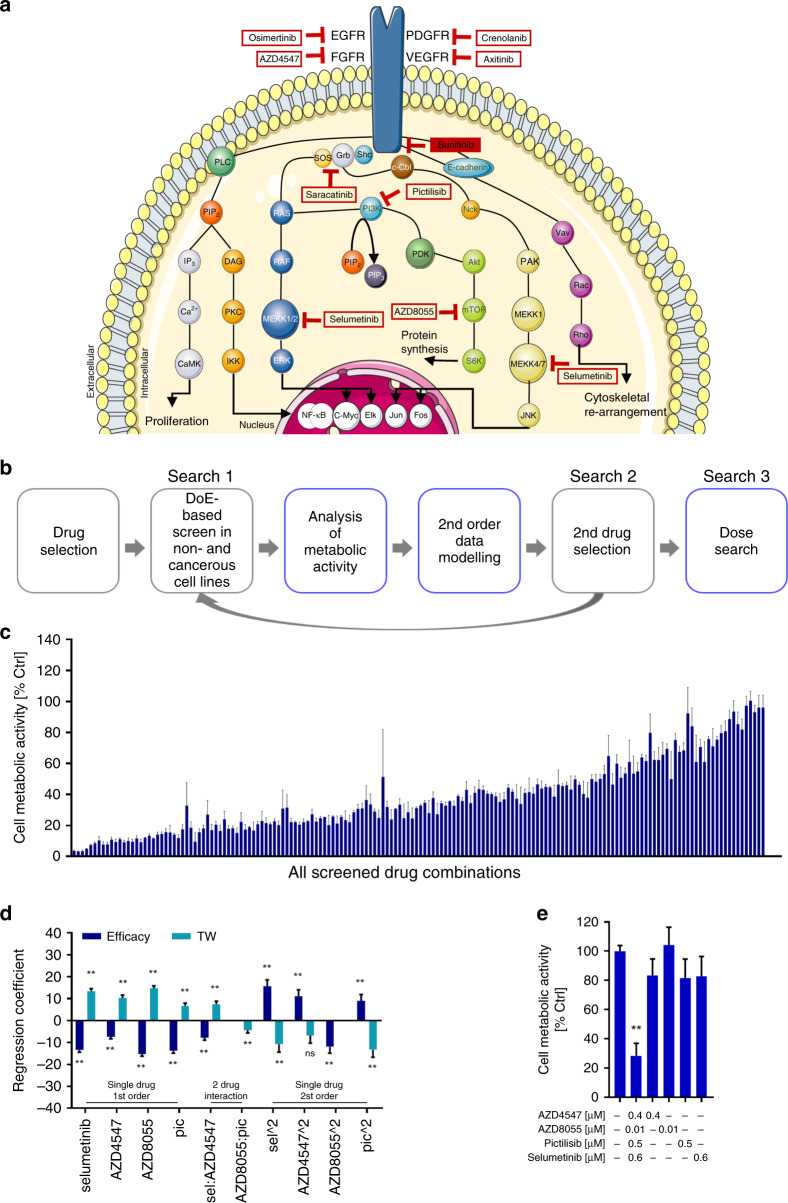
Drug optimisation platform and experimental validation of optimised drug combinations in A489-ST cells. **a** General scheme of selected drugs and their targets within important cell signalling pathways. **b** The pipeline of the TGMO search to find optimised low-dose drug combinations. **c** Within three searches, over 200 drug combinations are experimentally tested; the results in cell metabolic activity inhibition are presented from the lowest to the highest efficacy. **d** Representation of the results of *search 3* visualising the best drug candidates for the final drug combination of A498-ST. Regression coefficients of the models of efficacy (blue) and the therapeutic window (turquoise) are presented. Significance is represented with **p < 0.01. **e** The activity of the final drug combination in cell metabolic activity (% Ctrl) and the monotherapies at corresponding doses. Error bars represent the standard deviation (*N* = 2–3). Significances of **p < 0.005 define the difference of the ODC treatment to the Ctrl and each corresponding monotherapy determined with one-way ANOVA with Tukey’s multiple-comparison test.

Starting with a set of ten small-molecule-based drugs that are well known and well-characterised, either FDA approved or in clinical evaluation or with existing clinical data (Supplementary Table S1), we performed an experimental search using a simple in vitro cell metabolic activity assay. The latter indirectly corresponds to cell viability.^25^ All selected compounds bind to different extra- or intracellular components of growth factor receptors (GFR) (Fig. 1a and Supplementary Table S1). The main targets are the EGFR, the FGFR and the VEGFR, as well as enzymes of the MAPK/Erk,^26^ PI3K/Akt^27^ and Grb2/Nck^28^ pathways. Three RCC cell lines, i.e. A498, Caki-1 and 786-O varying in their origin and mutation status were used (Supplementary Table S2). In order to mimic the clinical situation where RCC patients receive sunitinib as the first-line therapy, we chronically treated cells with 1 µM sunitinib in order to induce insensitivity to sunitinib. The response of cells to sunitinib treatment was experimentally verified every 2 weeks, until cells became unresponsive to sunitinib (Supplementary Fig. S1A). Moreover, sunitinib-treated (ST) cells showed clear accumulation of sunitinib in lysosomes, in agreement with previously reported studies^29–32^ (Supplementary Fig. S1B).

In the first step, single-drug dose–response curves were generated for cell metabolic activity in all cell lines (Supplementary Fig. S1C). Subsequently, the following doses were selected for use in the TGMO-based search (Fig. 1b): (i) dose where 20% of the maximal response was observed; (ii) a dose representing half of the concentration of dose (i) and dose 0 (ED_0_), where this drug was absent in the mixture. Moreover, the maximum doses of all compounds were limited to clinically attainable doses based on the calculation of maximum drug plasma doses obtained in patients in clinical applications. The optimisation was accomplished by performing a series of orthogonally designed multidrug experiments (Fig. 1b, c) simultaneously in RCC and non-cancerous cell lines (human embryonic kidney cells, HEK-293T) (Fig. 1d), developing a therapeutic window (TW, i.e. % control of non-malignant cell metabolic activity—% control of cancer cell metabolic activity) for efficacy. Data obtained from these experiments (Fig. 1c) were used to build second-order linear regression models, which enabled the analysis and the elimination of non-synergistic drugs for a subsequent drug-dose search (Fig. 1d). In each regression model, an estimated regression coefficient was generated for significant terms. The latter were grouped as “single-drug first order”, “two-drug interaction” and “single-drug second-order” terms. They describe the contribution of each drug as an individual agent and as a drug pair to the overall activity of the drug combinations.

Appropriate data and model verification methods were implemented to ensure the accuracy and reliability of model-based predictions, including the verification of the main assumptions of the linear regression models i.e. weak exogeneity, linearity, constant variance, independence of errors and lack of multi-co-linearity. This was achieved globally, for each cell line, through the analysis of the coefficient of multiple determination (*R*^2^) and the plot of observed vs. fitted data points, and by residual analysis through visualisation of residual plots, Cook’s distance plot, normal Q–Q plot and residual histogram (Supplementary Fig. S2).

In *search 1*, 91 drug combinations were tested experimentally. Based on the stepwise regression analysis, three drugs were eliminated from further search due to the lack of synergy or antagonism (axitinib, crenolanib and icaritin for A498-ST cells). In the following *search 2*, we tested a set of 50 drug mixtures composed of up to seven drugs along with a set of 25 four-drug mixtures. Finally, *search 3* was performed to evaluate the most promising four-drug combinations identified in *search 2*.

ODC in sunitinib-naive and sunitinib-ST cells is listed in Table 1. Cell line-specific ODCs impaired cell metabolic activity, after a single treatment, with 66–72% efficacy, respectively. Corresponding monotherapies were relatively inactive (Fig. 1e for A489-ST cells and Supplementary Fig. S3 for other cells). Data analysis releveled the cell line-specific drug–drug interactions. As shown in the example of A489-ST cells, the ODC was composed of AZD4547, AZD8055, pictilisib and selumetinib. The main synergistic activity was found between AZD4547 and selumetinib (Fig. 1d). The biological measures of drug interactions for other cell line-specific ODCs are presented in Supplementary Fig. S3A–F.

We also performed in silico analysis of possible alternative ODC mechanisms of action, see Supplementary Information. Using drug concentrations applied in each cell line-specific ODC, we minded ProteomicsDB repository for additional protein targets with an effective inhibition score of ≥50% and retrieved. Interestingly, for A489-ST-specific ODC at doses applied, targets for drugs used in the combination (Supplementary Fig. S4A, upper-left graph) do not seem to play a role in a particular network (Supplementary Fig. S4A, bottom-left graph). Since the potential targets might be extended with first- and second-order interactors, we added up 20 proteins per each order interaction using STRING. As a result, we observed the induction of tight clusters and multiple interactions. For example, the FGFR1 connection with other networks affecting e.g. cyclin family, eukaryotic translation initiation factor 2-alpha kinase (EIF2AK) proteins and other essential survival factors such as PLK1, might be lethal in most of the cells when knocked down (Supplementary Fig. S4A, right graph). Based on gene ontology (GO) analysis, the biological processes behind the targets were classified using beneontology.org as mostly related to phosphorylation of MAPK signalling regulation. The results for other cell lines are presented in Supplementary Fig. S4B–F.

It is important to note that all RCC-specific ODCs contained two core compounds, i.e. AZD4547 and pictilisib, whereas 5 out of 6 drug mixtures also contained AZD8055. Therefore, this drug combination seems to be transversally potent in all tested RCC subtypes. Moreover, the A498-ST-specific ODC represented the best overall activity in all RCC cell lines with the lowest doses (Supplementary Table S3). Therefore, we selected the A498-ST-specific ODC as the most promising one, and present the respective results in the main figures, whereas the results for the other cell lines are shown in the Supplementary Material.

**Fig. 2:**
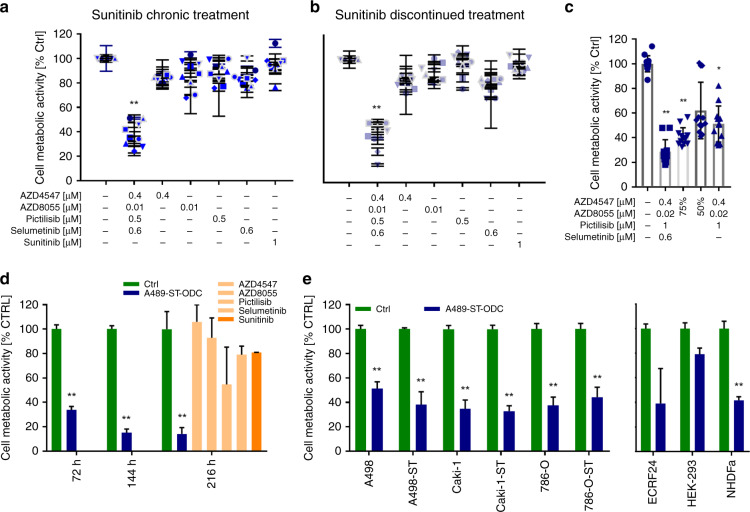
Activity of A498-ST-specific ODC in various conditions. Efficacy of the ODC and corresponding monotherapies over a period of 70 days after chronic (**a**) or discontinued (**b**) treatment with 1 µM sunitinib. **c** Decreased efficacy of the ODC by reducing the dose to a final concentration of 75% or 50%, or by removing one drug (selumetinib). **d** Cell metabolic activity inhibition after prolonged incubation of A498-ST cells (72, 144 and 216 h) with the ODC and corresponding monotherapies, as well as sunitinib used as positive control. **e** Cross-validation of the optimised drug combination established for A498-ST in other RCC and non-cancerous cell lines. Error bars represent the standard deviation (cell metabolic activity measurements, *N* = 2–3). Significances of *p < 0.01 and **p < 0.005 define the difference of the ODC treatment to the Ctrl and corresponding monotherapies determined with one-way or two-way ANOVA with Tukey’s post hoc test.

Since RCC is a highly vascularised tumour, targeting tumour endothelium may potentiate the overall treatment effect. Therefore, we optimised a four-drug combination specific to the human endothelial cells (ECRF24). This drug combination was composed of axitinib, pictilisib, saracatinib and selumetinib. Moreover, the incubation of ECRF24 cells with A498-ST-specific ODC, led to 61% of reduction of cell metabolic activity (Fig. 2e).

### ODCs maintain the activity in time in chronically treated cells with sunitinib

Stable acquired insensitivity to 1 µM sunitinib was obtained after multiple weeks (cell line specific) of continuous retreatment of cells. To monitor the kinetics of this insensitivity in time, we separated the cells into two subpopulations, i.e. (i) cells exposed to chronic treatment where sunitinib was maintained over a period of 70 days, and (ii) cells in which the sunitinib treatment was discontinued. Over this period, the cells were treated once weekly, and the cell metabolic activity readout was performed. ODC activity persisted, inhibiting cell metabolic activity up to 80% in a subpopulation (i), see Fig. 2a for A498-ST cells and Supplementary Fig. S5A for other cell lines, as well as for (ii), see Fig. 2b for A498-ST cells and Supplementary Fig. S5B for other cell lines. This suggests that the mechanism of action of ODCs was not impaired by the presence of sunitinib.

The synergistic strength and the efficacy of the combination were evaluated in various ways. First, we evaluated the power of the final optimised four-drug combination for A498-ST cells in two ways. First, by reducing the dose of the four-drug combination by 25 and 50%, which subsequently reduced the efficacy in a linear fashion by 10 and 30% compared with the original ODC (Fig. 2c). Second, we removed the one drug that would add the least synergy to the overall efficacy of the drug combination, in this case, selumetinib, which resulted in a 20% decrease in cell metabolic activity.

Since in clinical conditions, the RCC patients are re-treated several times with a treatment regimen, which leads to an enhanced effect, we re-treated A498-ST cells three consecutive times for 72 h with the readout performed for 72 h, 144 h and 216 h post the first incubation, respectively. The overall efficacy of the ODC treatment increased specifically with the second treatment time point from 70 to 85% (Fig. 2d).

After establishing a cell line-specific ODC, we validated the efficacy in other RCC cell lines, as well as non-cancerous cell lines of different origin (HEK-293T and NHDFα, see Supplementary Table S2). In all RCC cell lines (A498, Caki-1 and 786-O), the cell metabolic activity was significantly diminished by 50–70%, independently of the sunitinib treatment (−ST) and oncogenic mutation status. Cell metabolic activity of epithelial cells and fibroblasts, making up a large proportion of the tumour stroma, was only partially influenced (50–60%), to a smaller extent than in the RCC cells. HEK-293T cells were insensitive to the ODC treatment (Fig. 2e). Each established four-drug combination exhibited the most potent and synergistic anticancer activity when applied at optimised doses, thus harming the non-cancerous populations only marginally.

### Starvation enhances the activity of ODCs in sunitinib-naive and pre-treated RCC cells

Following metabolic differences between non-cancerous and cancerous cells, it is known that cell survival depends on the supply of diver nutrients.^33^ Therefore, it is possible to induce short-term starvation of cancer cells, while maintaining healthy metabolism in non-cancerous tissue, and potentiate the effect in cancer cells.^34^ Our results show that starvation of cells by serum deprivation enhances the power of the ODC treatment by 10–15%, resulting in an overall efficacy of 94%, in A498 and A498-ST cells, respectively (see Supplementary Fig. S6 for all cell lines). Access to glucose seems to be of less importance, which is in agreement with other reports.^33,34^

### ODCs are active in heterotypic 3D co-cultures

**Fig. 3:**
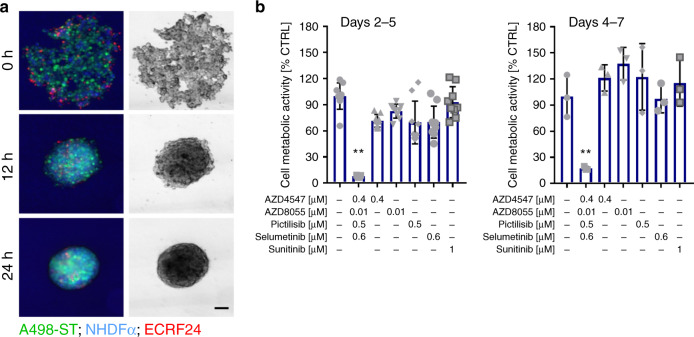
OCD activity in 3D co-culture models. **a** Representative images of heterotypic 3D co-culture spheroids composed of cell tracker-stained A498-ST cells (green), NHDFα (blue) and ECRF24 cells (red), and the 3D spheroid development over the first 24 h. Scale bar represents 150 µm. **b** Spheroid cell metabolic activity in A498-ST 3D co-cultures after treatment with Ctrl, ODC, corresponding monotherapies or 1 µM sunitinib (positive control). The results were obtained after administering each treatment for 72 h at two treatment schedules, i.e. (i) day 2–5 after spheroid formation (left graph), or (ii) day 4–7 after spheroid formation (right graph). Error bars represent the standard deviation (metabolic activity measurements, *N* = 2–3). Significances of **p < 0.005 define the difference of the ODC treatment to the Ctrl and each corresponding monotherapy determined with an ordinary one-way ANOVA with Tukey’s post hoc test.

Subsequently, we validated the activity of the ODCs in heterotypic 3D co-culture systems.^24^ They were composed of 70% RCC cells, 10% endothelial cells (ECRF24) and 20% fibroblasts (NHDFα), in order to mimic the human RCC tumour composition. The cell suspension was supplemented with 2.5% Matrigel to deliver the extracellular matrix (ECM), and also to promote ECM production by the cells themselves.^24^ Staining of each cell population within the 3D co-culture with a cell tracker dye of A498-ST cells in green, NHDFα in blue and ECRF24 in red visualised the first 24 h of spheroid formation (Fig. 3a and Supplementary Videos S1–S2). The results were obtained administering each treatment for 72 h at two treatment schedules, i.e. (i) day 2–5 after spheroid formation (Fig. 3b, left graph), or (ii) day 4–7 after spheroid formation (Fig. 3b, right graph). Representative spheroid bright-field images recorded in consecutive spheroid growth days, are shown in Supplementary Fig. S7A. The ODC treatment efficacy, when treating already after 48 h of culturing (day 2–5 treatment schedule), in the 3D A489-ST co-culture system reached 93% exceeding the measured efficacy of 70% in the 2D culture system (compare Fig. 1e). The ODCs of the other cell lines were similarly more effective in 3D co-cultures than in 2D, i.e. in 786-O cells (94%) and 786-O-ST cells (91%), and to a lesser extent in Caki-1 and Caki-1-ST cells (Supplementary Fig. S7B). Independently of the treatment schedule, sunitinib treatment remained ineffective. Therefore, our 2D-identified cell line-specific ODCs could be translated into more complex heterotypic 3D systems.

### Cell morphology after cell line-specific ODC treatment

**Fig. 4:**
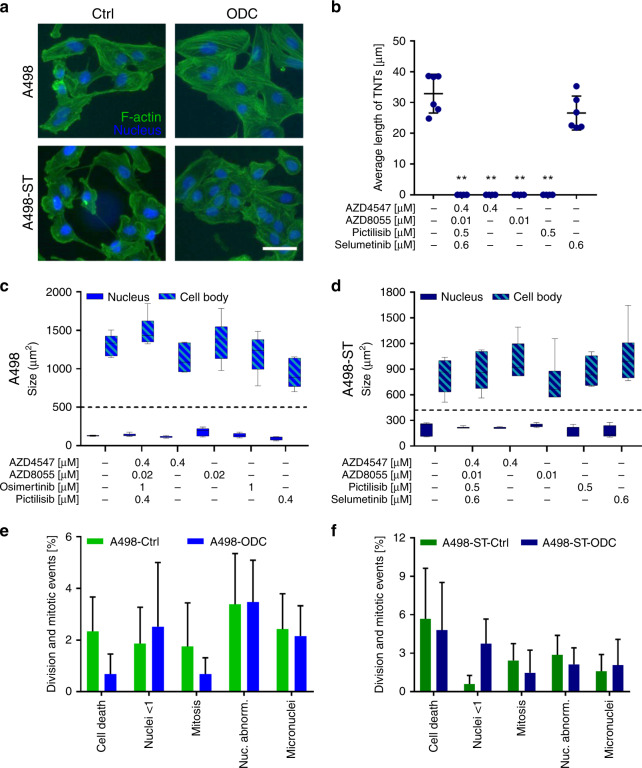
Functional analysis of the cellular morphology after treatment. **a** Representative images of A498 and A498-ST cells stained for f-actin (phalloidin, green) and nuclei (Dapi, blue). In total, 19.000 cells/well were seeded, incubated with the ODC or corresponding monotherapies and imaged after 24 h. Scale bar represents 20 µm. **b** Quantification of the presence and average length of connected and non-connected tunnelling nanotubes after treatment. **c**, **d** The size of the cell body (striped, above dashed line) and nucleus (full, below dashed line). **e**, **f** Quantified mitotic abnormalities plotted for Ctrl and ODC-treated A498 and A498-ST cells. Error bars represent the standard deviation (*N* = 2, 6 images of two biological repeats). Significances of **p < 0.005 define the difference of the ODC treatment to the Ctrl determined with paired *t* test.

To identify possible changes in the cellular morphology after ODC and corresponding monotherapy treatments, the RCC cells were stained for f-actin (GDP-labelled phalloidin, green) and nuclei (Dapi, blue). The morphometric analysis of RCC cells 24 h post ODC treatment (Fig. 4 and Supplementary Fig. S8) was performed and showed heterogenic results. We noted morphological changes between A498 and A498-ST ODC-treated cells vs. control (sham-treated cells), see representative images in Fig. 4a, where the alterations of the actin cytoskeleton are depicted for A498-ST cells, defining the cell body size, number of cell–cell connections and the nucleus size. The ODC treatment induced the formation of stress fibres visualised by polymerised f-actin (Fig. 4a, ODC). Moreover, ODC treatment increased the cellular size (Fig. 4a, c and d) and induced cell clustering (Fig. 4a and Supplementary Fig. S8). Analysis of other cell lines can be found in Supplementary Fig. S8D, E.

An analysis of nuclear events, such as the number of multinuclear cells, mitotic cells, micronuclei or other mitotic abnormalities in ODC-treated and control cells, was carried out. The results were cell-line dependent. No significant differences were observed in A489-ST cells (Fig. 4e, f), whereas a significantly increased number of multinuclear cells was observed in Caki-1-ST cells (Supplementary Fig. S8). Notably, we observed the presence of tunnelling nanotubes (TNTs) in A498-ST cells, which are actin-containing membrane protrusions (>10 µm), which are known to play an essential role in long-range intercellular communication. Treatment with particular monotherapies or the ODC impaired the formation of these protrusions as presented by the average TNT length (Fig. 4b and Supplementary Fig. S8).

### ODCs modulate cell cycle and induce cell death in RCC cells

**Fig. 5:**
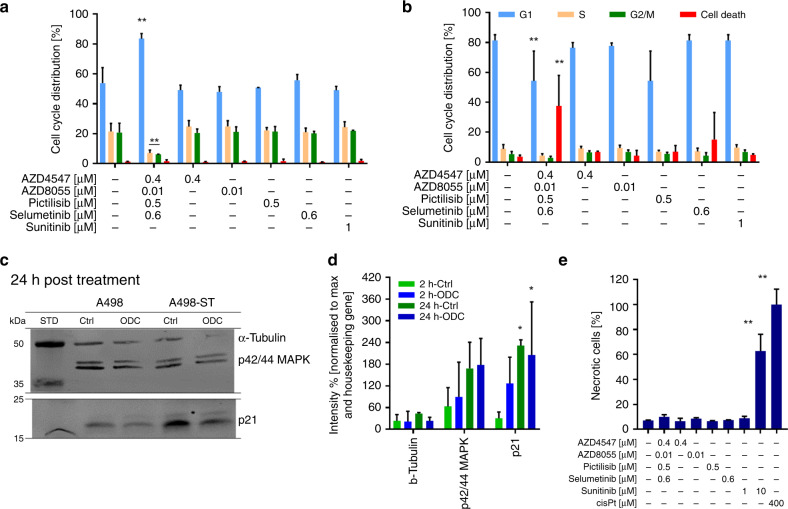
Abnormalities of cell division and induction of cell death. **a**, **b** Cell cycle analysis with flow cytometry analysis of FxCyclePI after 24-h (**a**) and 72-h (**b**) incubation with treatments presenting the cell cycle distribution within G1, S, G2/M phase and cell death. **c** Western blot visualisation after 2 h and 24 h of treatment of the expression of p42/44MAPK and p21 in comparison with the housekeeping protein α-tubulin. **d** Quantification of western blot results. **e** Necrosis induced after treatment with ODC, corresponding monotherapies and positive controls (1 µM sunitinib, 10 µM sunitinib and 400 µM cisplatin). Error bars represent the standard deviation (*N* = 2, 6 images of two separate experiments). Significance of **p < 0.005 defines the difference of the ODC treatment to the Ctrl determined with a paired *t* test.

Investigating the mechanism of action of ODCs, we looked at the cell cycle, necrosis and senescence. We monitored cell cycle distribution and the frequency of cell death by flow cytometry using propidium iodide staining. Exposure of A498-ST cells to the ODC for 24 h resulted in a significant arrest in the G1 phase (≥30% cells arrested in G1 vs. control, Fig. 5a), consequently leading to cell death (38%) measured after 72 h of cell incubation with ODC (Fig. 5b). Cell cycle results for other cell lines are presented in Supplementary Fig. S9.

Further, we evaluated the expression of MAPK (4695S) and the cyclin-dependent kinase (CDK) inhibitor protein p21 (2947S), molecules known for playing a role in cell cycle regulation and senescence. Western blot was performed 2 h and 24 h post ODC treatment (Fig. 5c and Supplementary Figs. 10, 11). Independently, the expression levels of MAPK did not change significantly, independent of the duration of the ODC exposure. No significant alterations between sunitinib-naive and A498-ST cells were noted. In contrast, the expression of p21 was significantly altered after ODC exposure in a time-dependent manner (Fig. 5c, d).

Using annexin V staining of ODC-treated cells, we demonstrated that neither treatment with the ODC nor the corresponding monotherapies induced necrosis in treated cells. Sunitinib at a concentration of 10 µM (63%), as well as 400 µM cisplatin (100%), used as positive controls, leads to a significant increase in necrosis (Fig. 5e).

Our data suggest that the mechanism of action of the ODC treatment might be due to immediate interference in pathways promoting the G1 phase. It seems that this particular blockade of the cell cycle further leads to the induction of apoptosis-mediated cell death.

## Discussion

In this study, we used the s-FSC technology to identify four-drug combinations specific to sunitinib-naive and sunitinib pre-treated RCC cells. We experimentally tested only approximately 0.4% of all possible drug combinations containing 2–10 drugs in the mixture to determine cell line-specific synergistic drug combinations. This was done simultaneously in non-cancerous cells, allowing analysis to be performed based on a therapeutic window that selected against drug toxicity. Other standard approaches would require millions of drug combinations to be tested in order to reach the same outcome.

Interestingly, our system identified cell-type-specific ODCs all composed of AZD4547 and pictilisib, known to target FGFR and phosphatidylinositol 3-kinase, respectively. FGFR signalling network consists of multiple pathways, such as ERK1/2 and PI3K/AKT/mTOR differentially activated, depending on the cellular context. Of note, the inhibition of the aforementioned pathways has previously been reported to play an instrumental role in the treatment of various cancer types.^35–37^ Although an in-depth investigation is required to identify the exact mechanism of action behind the drug–drug interaction and observed ODC activity, we can speculate that not only the known targets are involved. Based on the protein-interaction mapping analysis at the concentrations used in each cell line, the activity of each cell line-specific ODC may also act as well via indirect higher-order network interactions (Supplementary Fig. S4). Similarly, we have recently discovered that crenolanib, a known PDGFR inhibitor, acts on tumour endothelial cells, a mechanism that is not always directly related to its designated targets.^38^ Since resistance to sunitinib is a frequent event in the clinical management of RCC, one way to overcome this challenge is the optimisation of high-order drug mixtures to kill drug-resistant cancer cells.^19,39^ Notably, treatment with relatively low-dosed ODCs was effective also in cells chronically pre-treated with sunitinib. Those ODCs were shown to be superior to non-optimised drug combinations (not shown) as well as to sunitinib. Our ODCs are active after multiple administration in sunitinib-treated cells, and their activity is even stronger under short-term starvation conditions (Supplementary Fig. S9). Repeated drug intake following a well-designed schedule seems to result in a promising treatment strategy.

Morphometric analysis of the cells chronically treated with sunitinib did not show hypertrophy as compared with sunitinib-naive cells. However, this phenomenon was observed by others in RCC cells treated with 10 µM sunitinib.^40,41^ This difference might be due to the fact that long-term treatment with a lower dose of sunitinib, in our case 1 µM, which is sequestered in lysosomes of the cell, does not lead to any cell skeleton adaptation. We noticed, however, the appearance of hypertrophy in Caki-1-ST cells after ODC- and the corresponding monotherapy treatment. The exact mechanism of this phenomenon is worth pursuing, and it could be related to the impaired migratory properties of these cells.^42^ Interfering with the EGFR–MAPK signalling pathway can lead to such morphologic changes by increasing cell–cell contacts profoundly stabilising focal adhesions.^43^ Therefore, it seems that the loss of motility may be induced by blocking EGFR–MAPK signalling, further modulating the cell-matrix and simultaneously enhancing cell–cell interactions.^42^

Malignant cells are capable of communicating with neighbouring cells, as well as the tumour microenvironment by forming tunnelling nanotubes (TNT).^44,45^ Intercellular exchange of organelles, vesicles and signalling molecules can occur, which mediates the passage of resistance and the insensitivity to small-molecule drugs.^46^ A498-ST (Fig. 4) and Caki-1, as well as Caki-1-ST cells (not shown), formed TNTs with an average length of ≤45 µm. Treatment with ODC destroyed already-existing tubes and blocked the formation of new connections. Our data suggest that applying the ODC blocks long-distance cell communication and interchange that might alleviate the aggressiveness of RCC and decelerate tumour progression.

The abnormal proliferation and cell cycle regulation are hallmarks of cancer cells.^47^ These disordered processes are influenced after short- and long-term treatment with cell line-specific ODCs. The cell division, in particular, the progression from G1 to S phase of the mitotic cycle is impaired. It appears that this blockade is the leading cause driving RCC cells into apoptosis. Exceptionally, our results revealed that Caki-1 cells die via necrosis after ODC treatment. Senescence-mediated cell fate via p21 and MAPK is not increased after 2 h or 24 h of ODC treatment. However, senescence appeared due to chronic sunitinib treatment shown by western blot.^48,49^

Another important finding of this study was that the potent anti-proliferative effects of the ODCs observed in simple 2D in vitro assays were also present in complex 3D co-culture systems where the crosstalk between RCC, fibroblasts and endothelial cells is present. In some cell lines, the activity of ODCs was even more potent in 3D conditions (Fig. 3 and Supplementary Fig. S7), which might be related to the different target expression as compared with 2D models.^50,51^

Since RCC is known to be chemo- and radio-resistant,^52,53^ current clinical treatment is based on the use of angiostatic targeted tyrosine kinase inhibitors, administered individually or in sequenced combinations.^54^ However, these drugs do not radically prolong the survival of RCC patients mostly due to the acquired drug resistance.^2,55,56^ As targeted drugs were originally aiming for angiogenesis inhibition, we performed a dedicated TGMO-based drug optimisation in human ECRF24 cells. The ODC found for ECRF24 cells, although containing drugs such as pictilisib, which was present in all cancer cell line ODCs, was less effective, only inhibiting approximately 50% in ECR24. Interestingly, it was also found that all RCC-cell line-specific ODCs were potent in ECs (Supplementary Table S3). That indicated that both anticancer and anti-angiogenic mechanisms constitute the global activity of the ODCs. This is an expected advantage, considering the intrinsic sensitivity of RCC for angiogenesis inhibitors.

Targeting various molecules up- and downstream of GFR signalling pathways is a common strategy in cancer treatment. One approach to treat RCC is to use multi-kinase inhibitors, i.e. sunitinib,^57^ pazopanib^58^ or cabozantinib.^59^ However, the doses applied are high, leading to enhanced off-target effects and the appearance of severe side effects. In the case of sunitinib, pazopanib and cabozantinib, the targets where sunitinib binds with the strongest affinity are located at an equal level within tyrosine kinase signalling. Combinations of drugs that have a specific targeting profile in different signalling pathways at lower doses will demonstrate a better safety profile, and are easier to adapt in a personalised fashion.^60^

Previous studies have shown the importance of combining drugs that interfere with molecules up-^61–63^ and downstream^64–66^ of a certain signalling cascade. However, the choice of targets and drugs is crucial and determines treatment success. Combinations of mTORC1 inhibitors with anti-VEGF agents have failed in the clinic the treatment of advanced RCC,^67^ because of inaccurate composition. Nonetheless, this does not exclude the benefit of combined blockade of these targets for validation in experimental or clinical settings.^68–70^

As a consequence, we suggest that our way of designing combinations of four drugs at low doses, inhibiting GFR signalling at multiple levels and within multiple pathways, is valuable in the development of innovative treatment options for RCC. We are confident that the ODCs validated through our study are of high quality, as it has been demonstrated that a triple combination of AZD8055, pictilisib and selumetinib can reduce tumour growth in vitro as well as in vivo settings.^66^ The potency of the anticancer efficacy resulted from an underlying synergy between mTORC1/2 (AZD8055), PI3K (GDC-0941) and MEK1/2 (selumetinib) inhibitors.^66^ In accordance with these studies, we are confident that our approach revealed ODCs with highly selective and synergistic anticancer activity.

We are aware that our model systems do not fully represent in vivo conditions. Several factors, such as drug metabolism, drug clearance, specific organ toxicities, the presence of stem cell involvement and immunity in the treatment response were not evaluated in this study. However, it has been demonstrated previously that a triple combination of AZD8055, pictilisib and selumetinib can reduce tumour growth in in vitro as well as in vivo settings. The potency of the anticancer efficacy resulted from an underlying synergy between mTORC1/2 (AZD8055), PI3K (pictilisib) and MEK1/2 (selumetinib) inhibitors.^66^ In accordance with these studies, we are confident that our approach revealed ODCs with highly selective and synergistic anticancer activity. Nevertheless, clear evidence for the efficacy and safety profile of the combination has to be gained from preclinical and clinical trials. Therefore, we are currently planning validation of the above-described results’ in vivo models of RCC where tumour angiogenesis and immune responses can be studied in more detail.

## Conclusions

Optimised drug combinations of four drugs applied at low doses, always containing AZD4547 and pictilisib, have been identified as an effective regimen that is synergistically toxic to RCC cells and non-toxic in non-cancerous cells, both in sunitinib-naive and sunitinib-treated cells. ODC treatment induced apoptosis and outperformed current standard RCC care, sunitinib. Further mechanistic studies and preclinical validation will guide conceivably to clinical evaluation.

## Supplementary information

Supplementary Informaiton

Supplementary Tables

Video S1

Video S2

## Data Availability

All data generated or analysed during this study are included in this published article and its Supplementary information files. The following are available online at the *British Journal of Cancer* website; Fig. S1: sensitivity to sunitinib treatment of sunitinib-naive and sunitinib pre-treated RCC cells and dose–response curves for all selected drugs, Fig. S2: data and model verification methods implemented to ensure the accuracy and reliability of model-based predictions in Search 1, Fig. S3: excerpt of the optimisation process in RCC cell lines and ECRF24 cells to define a specific low-dose combination, Fig. S4: the network of the targeted proteins by the ODC, Fig. S5: maintenance of ODC activity following chronic or discontinued sunitinib treatment, Fig. S6: efficacy of the ODC treatment as a function of the nutrient supply, Fig. S7: efficacy of the ODC treatment in heterotypic 3D co-cultures, Fig. S8: induced abnormalities of cellular morphology after treatment, Fig. S9: treatment- and time-dependent alterations of the cell cycle and evaluation of necrotic events, Fig. S10: full-length unprocessed western blot results of MAPK and p21 in RCC cell lines after 2  h of treatment, Fig. S11: full- length unprocessed western blot results of MAPK and p21 in RCC cell lines after 24 h of treatment; Table S1: selected drugs and their targets, Table S2: characteristics of the cell lines used, Table S3: cross-validation of all RCC-specific ODCs; Videos S1–S2: 3D co-culture spheroid formation containing A498-ST cells.
