# Supplementary material for: Identification of low-dose multidrug combinations for sunitinib-naive and pre-treated renal cell carcinoma
Source: Br J Cancer. 2020 May 22;123(4):556–67. doi: 10.1038/s41416-020-0890-y (PMC7435198; doi:10.1038/s41416-020-0890-y)
Supplement: Supplementary file 1 — Supplementary Informaiton [file 41416_2020_890_MOESM1_ESM.docx]

**Rausch *et al*. Supplementary material**

**Identification of low-dose multidrug combinations for sunitinib naive and pre-treated renal cell carcinoma**

**Magdalena Rausch^1,2^, Andrea Weiss^1^, Joanna Achkhanian^1^, Andrei Rotari^1^, Patrycja Nowak-Sliwinska^1,2,^***

^1^Molecular Pharmacology Group, Institute of Pharmaceutical Sciences of Western Switzerland,1 Rue Michel-Servet, 1211, Geneva 4, Switzerland;

^2^Translational Research Center in Oncohaematology, 1 Rue Michel-Servet, 1211, Geneva 4, Switzerland

**Supplementary Information**

**Supplementary Figures S1-S11**

**Supplementary Tables S1-S3**

**Supplementary Videos S1-S2**

**Supplementary Information**

*Protein network visualization*

To examine potential interactions of genes involved in ODC activity, we applied the Search Tool for the Retrieval of Interacting Genes (STRING) to predict the protein-protein interaction networks. Predicted target profile list for each cell line specific ODC was created in ProteomicsDB with effective inhibition threshold for visualization of 50% transferred to [string-db.org](http://string-db.org) (v.11.0). Using the **‘**Analytics Toolbox**’** function, dose-dependent

protein-drug interactions was mined. and proteins reported with an effective inhibition of **≥**50% with this concentration of drugs in the ODCs were subsequently included for further analysis. The protein-protein interaction network was created (with confidence 0.4). In the next step, 1^st^ and 2^nd^ shell interactors, with up to 20 interactors per protein was added. Based on geneontology (GO) analysis (beneontology.org) the biological processes behind the targets were classified.


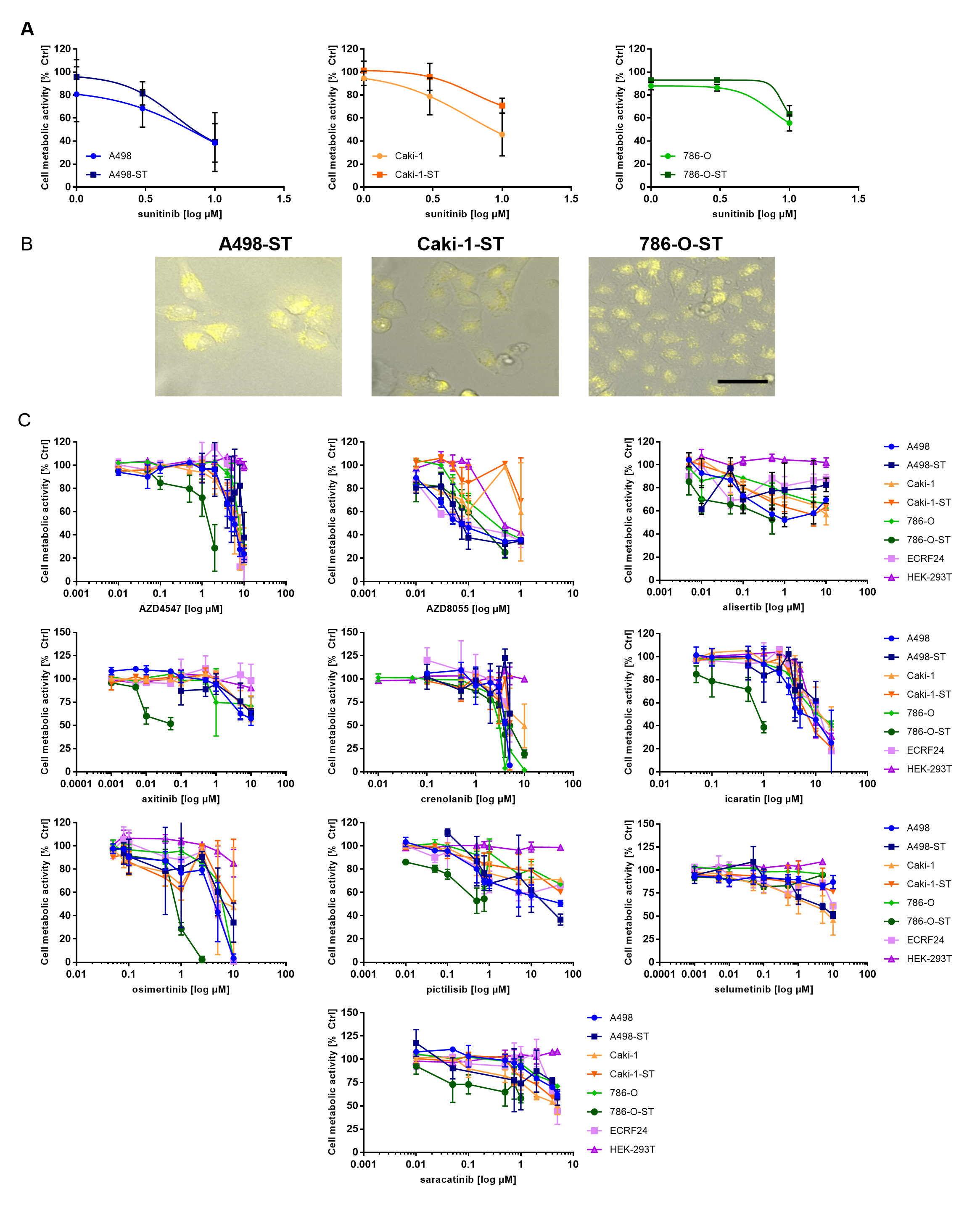


**Supplementary Figure S1: Sensitivity to sunitinib treatment of sunitinib-naïve and sunitinib-pre-treated RCC cells and dose-response curves for all selected drugs.**

**A**) Dose-response curves of log-transformed doses of sunitinib-naïve and sunitinib-pre-treated RCC cells. **B**) Representative pictures of all sunitinib pre-treated cells demonstrating the accumulation of sunitinib in lysosomes. Scale bar representing 20 µm. **C**) Dose-response curves for each compound of the primary selection in each cell line (Graphpad Prism^®^) (N =3). Error bars stand for the standard deviation.

**
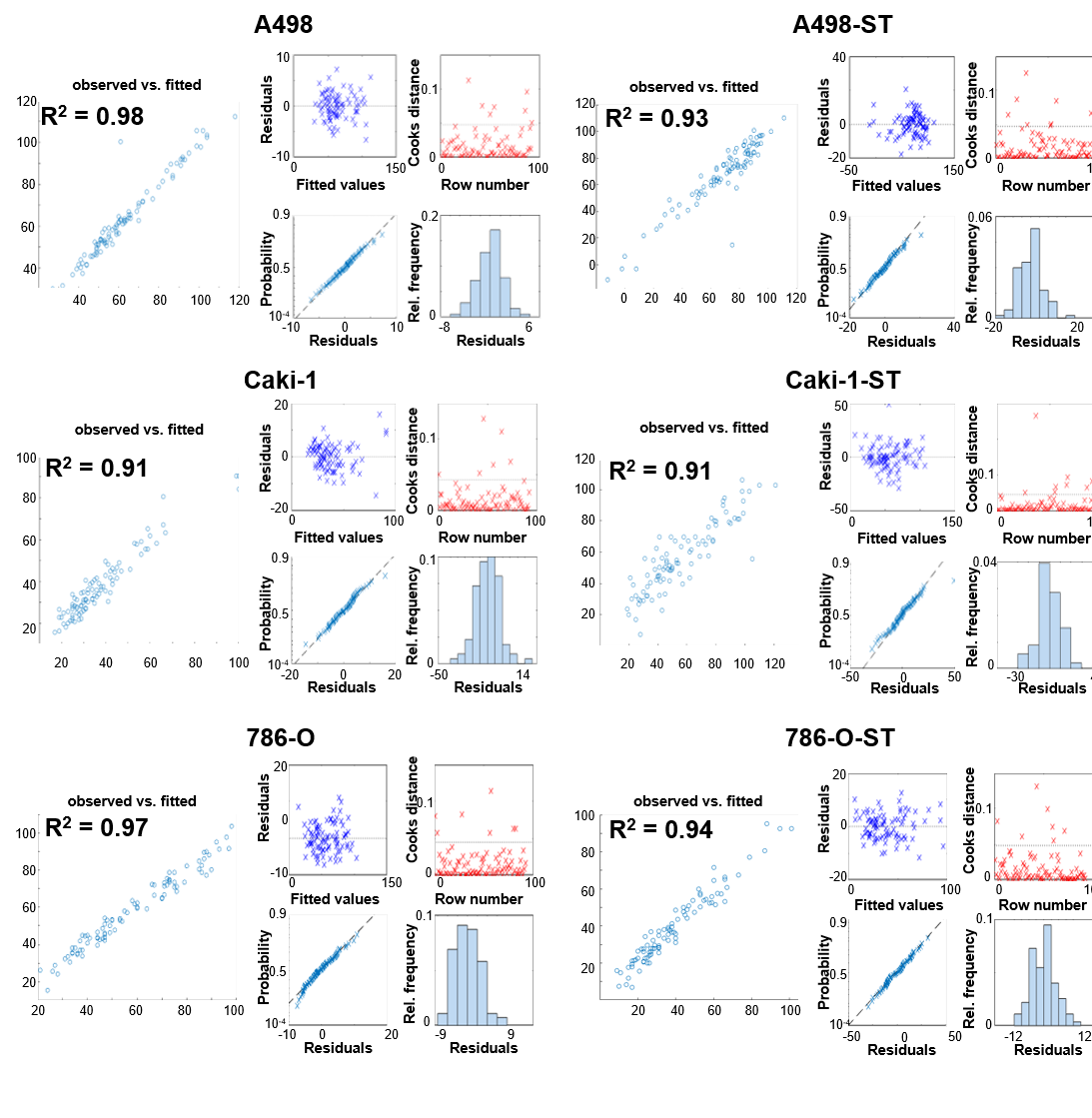
**

**Supplementary Figure S2: Data and model fitness analysis to evaluate accuracy and reliability of model-based predictions in Search 1.**

Analysis of the coefficient of multiple determination (R2) determined by residual analysis of observed vs. fitted data points. Visualization of residual plots, Cook’s distance plot, normal Q-Q-plot, and residual histogram. Statistical indicators of the goodness of each fit for the search 1 in all RCC cell lines.

**
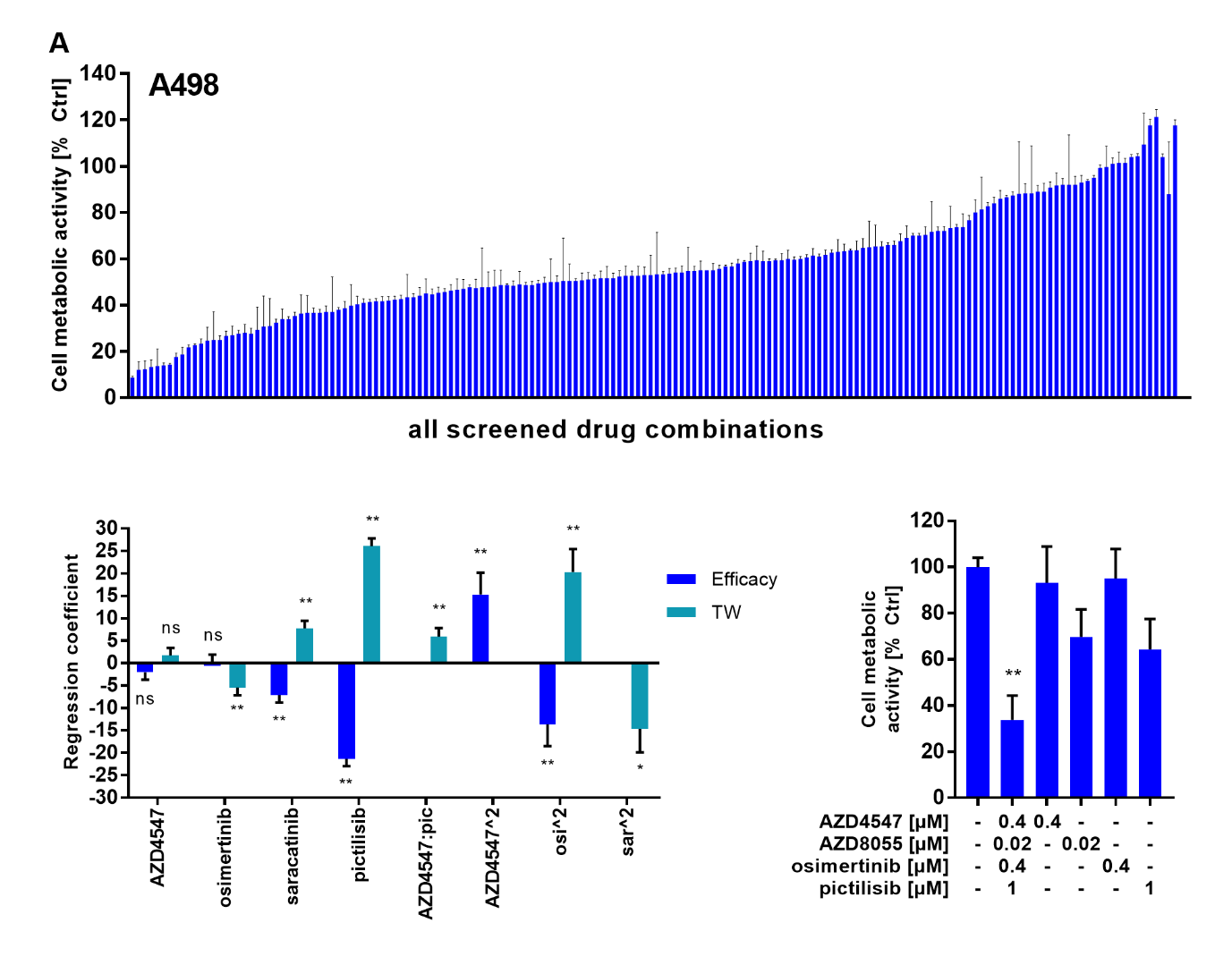
**

**
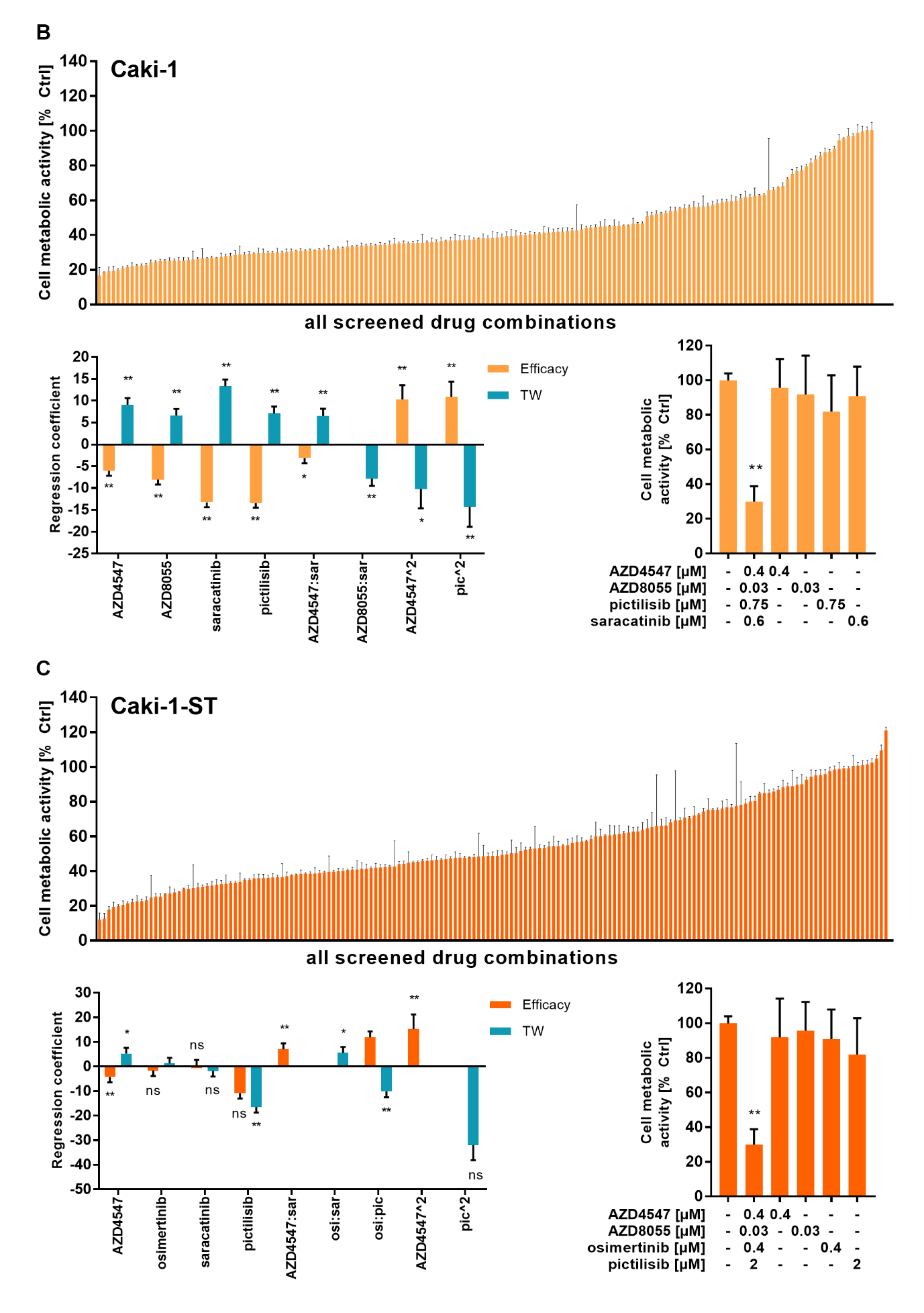
**

**
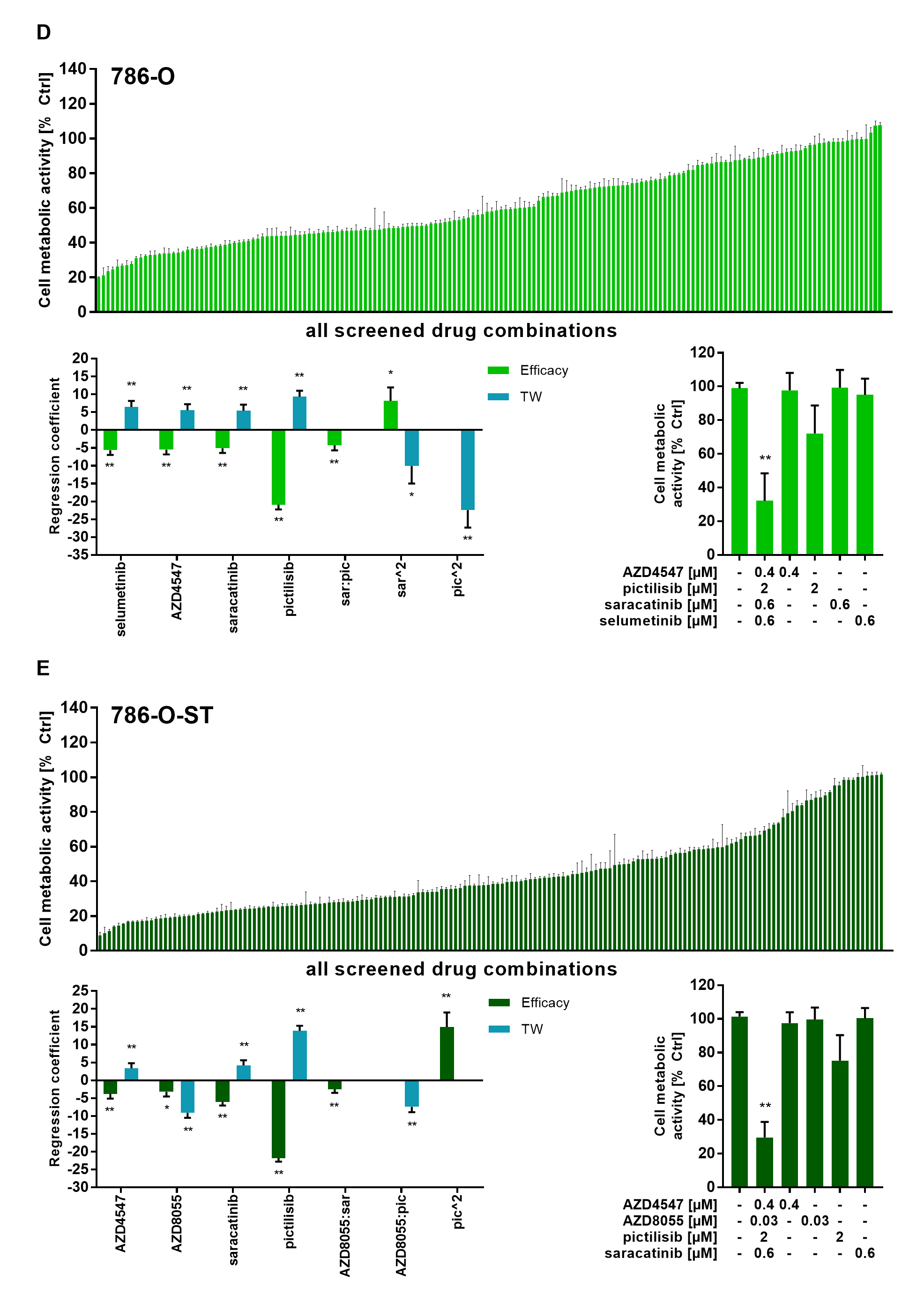
**

**
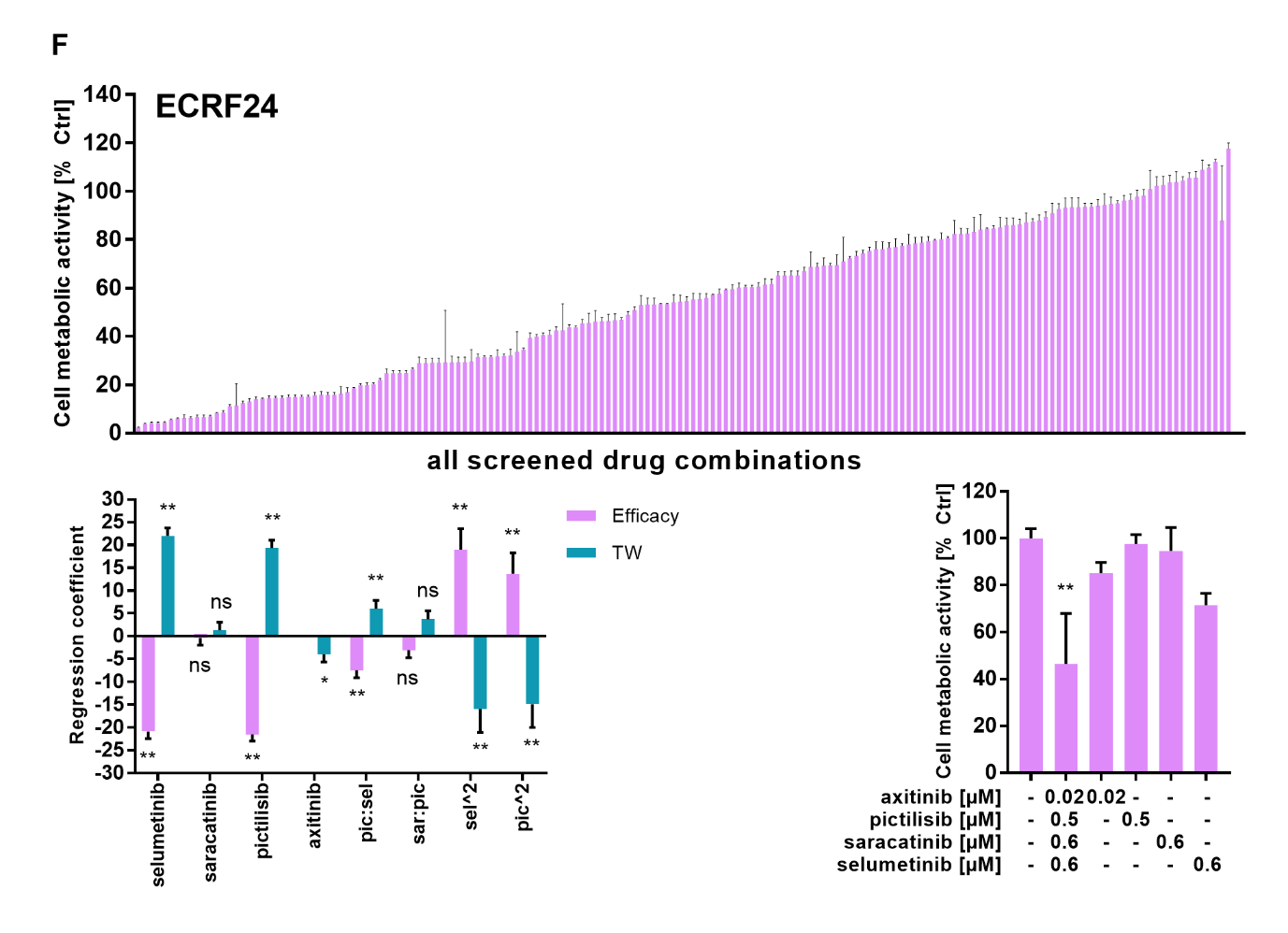
**

**Supplementary Figure S3: Excerpt of the optimization process in RCC cell lines and ECRF24 cells to define a specific low-dose combinations**

Three graphs per cell line **A**) A498, **B**) Caki-1, **C**) Caki-1-ST, **D**) 786-O, **E**) 786-O-ST and **F**) ECRF24 depict in short an overview throughout the whole screening process. The efficacy of all screened drug combinations within three searches is presented in the top graph. Bar graphs are sorted from highest to lowest efficacy. Representation of calculated regression coefficients (bottom left) in regard to the therapeutic window of the best drug candidates for a final ODC. The activity of the ODC (bottom right) and the corresponding monotherapies at corresponding doses. Error bars represent the standard deviation (metabolic activity measurements, N =3). Significances of *p < 0.01 and **p < 0.005 define the difference of the ODC treatment to the Ctrl and each corresponding monotherapy determined with ordinary one-way ANOVA (top), regression analysis (bottom left) and two-way ANOVA (bottom right) with Tukey’s post hoc test.

**A**

**
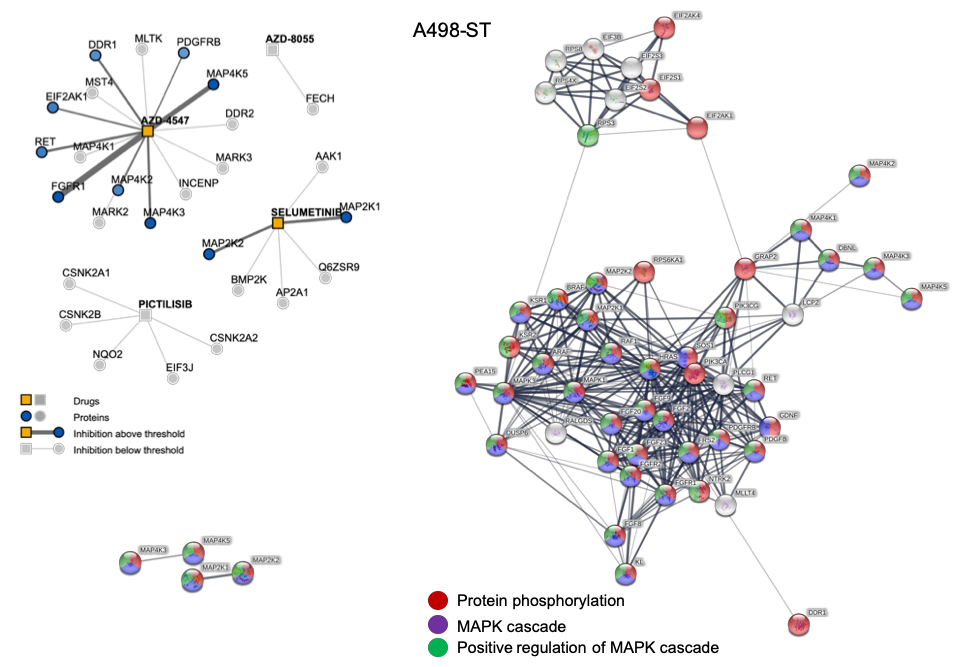
**

**B**

**
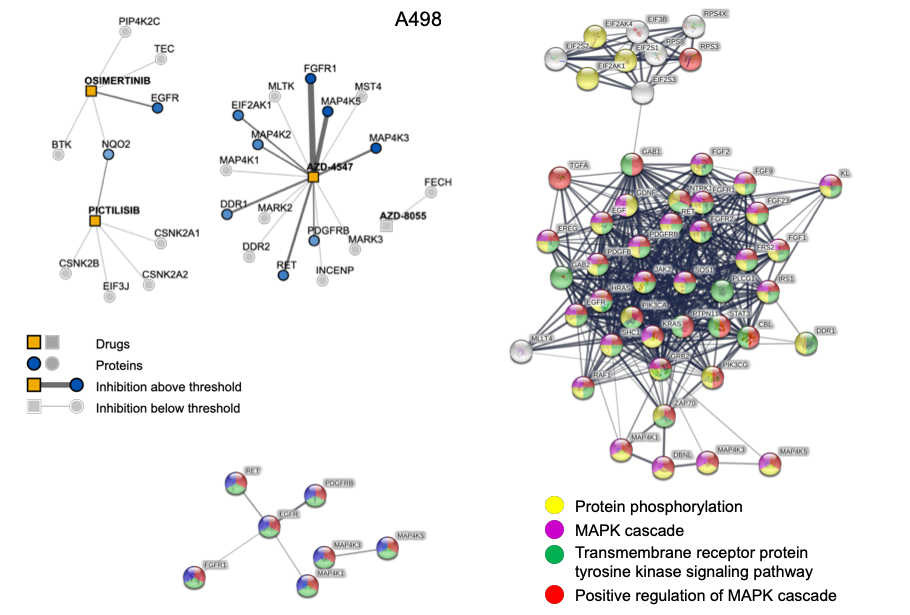
**

**C**

**
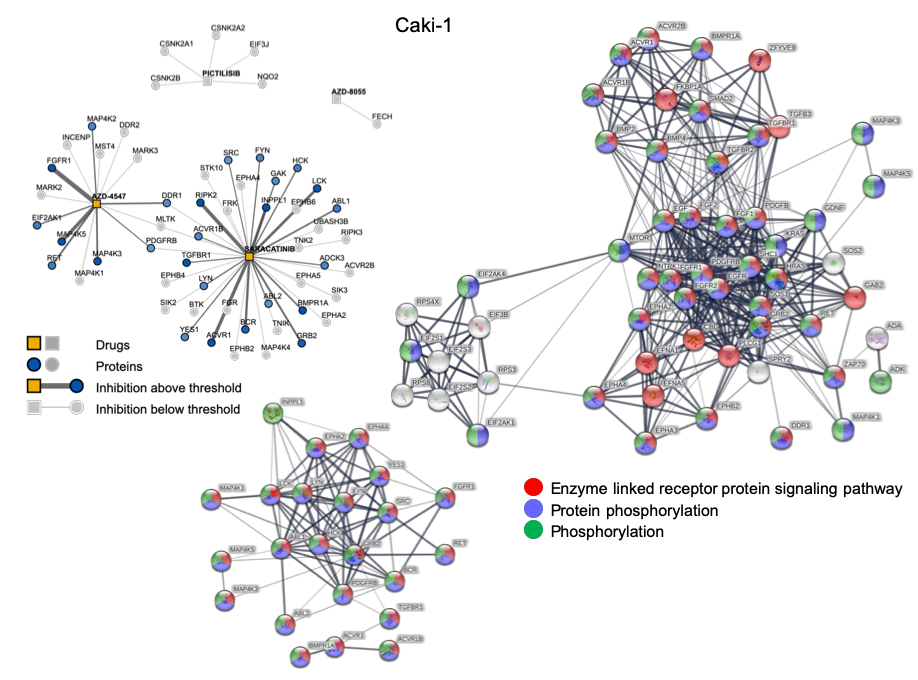
**

**D**

**
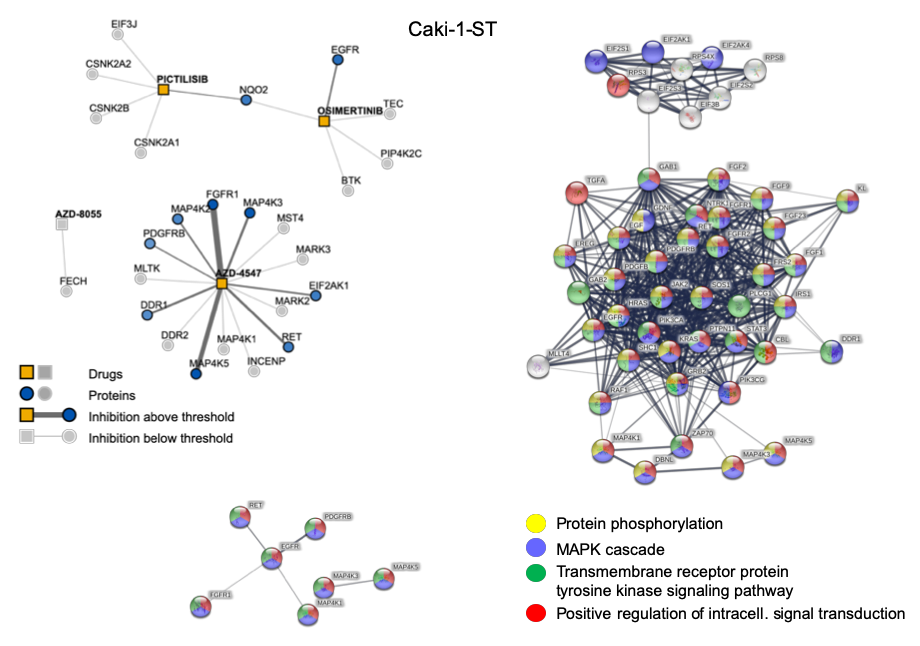
**

**E**

**
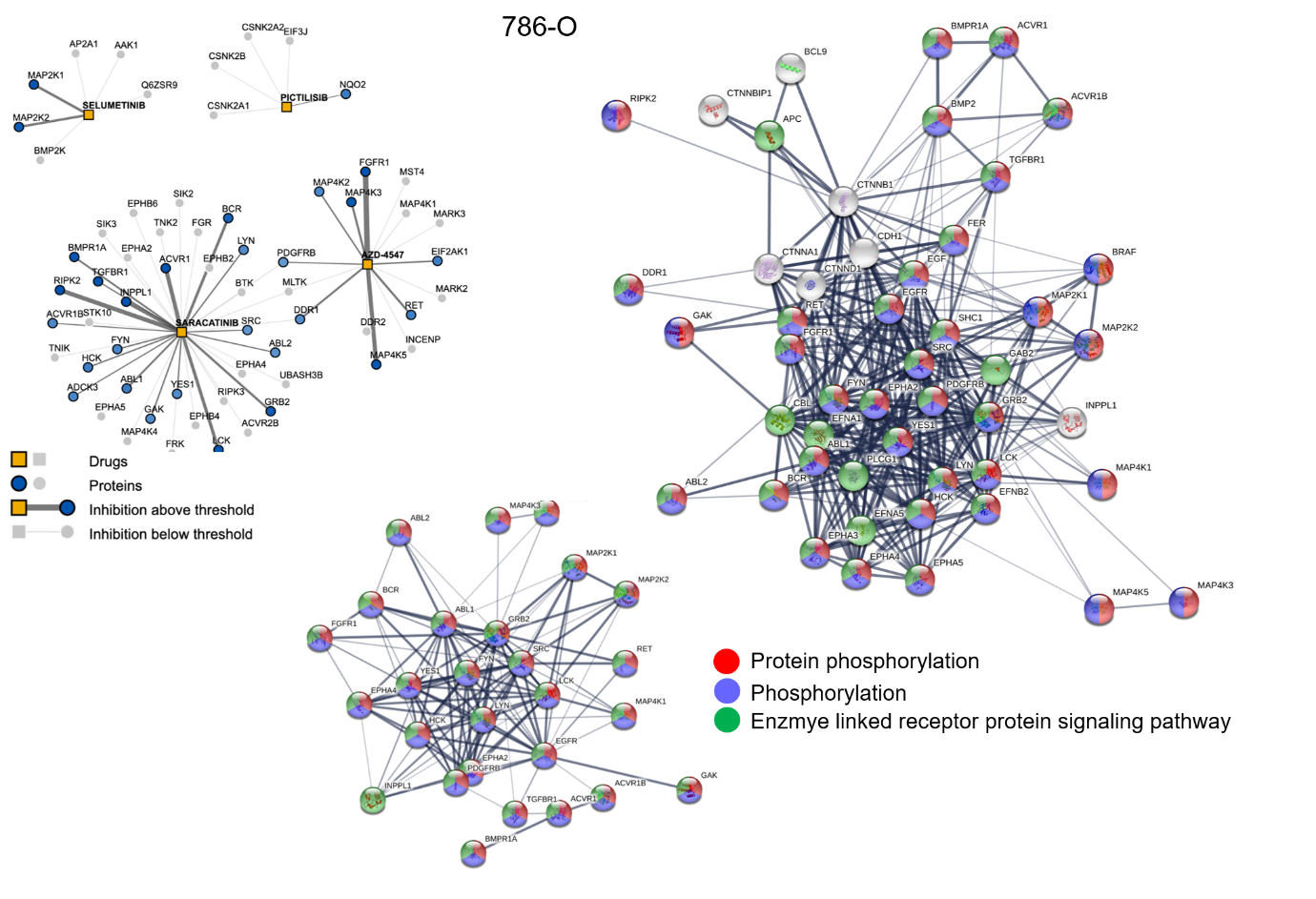
**

**F**

**
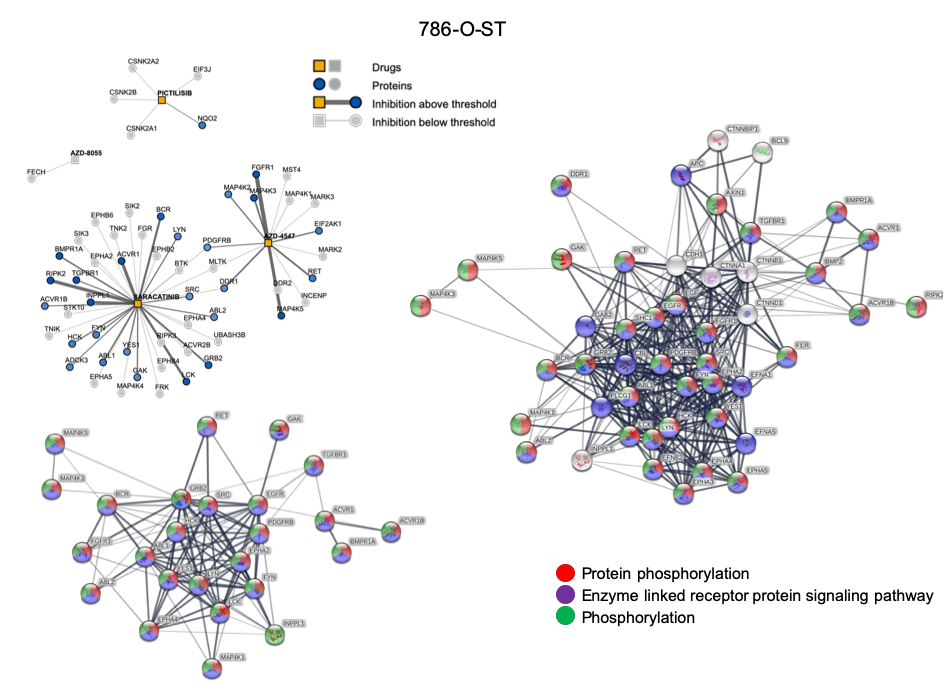
**

**Supplementary Figure S4: In silico analysis of the network of the targeted proteins by the ODC.**

**A**) A489-ST, **B**) A489, **C**) Caki-1, **D**) Caki-1-ST, **E**) 786-O, **F**) 786-O-ST.

**Top left:** The cell-line specific ODC concentrations were used to retrieve the predicted targets from proteomicsDB using effective inhibition threshold of 50%. Note multiple cases of inhibition below the threshold.

**Bottom left:** Illustration of the network using STRING of the targeted proteins by the ODC and after the network extension with first and second order interactors (Right). Less than 20 interactors was selected for the first and second order interactors. Interaction score was set at 0.4. Disconnected nodes were removed from the network. The line thickness indicated the strength of data support.The network view predicted the associations between proteins from the regulated genes involved in color-coded GO-based biological processes.

**
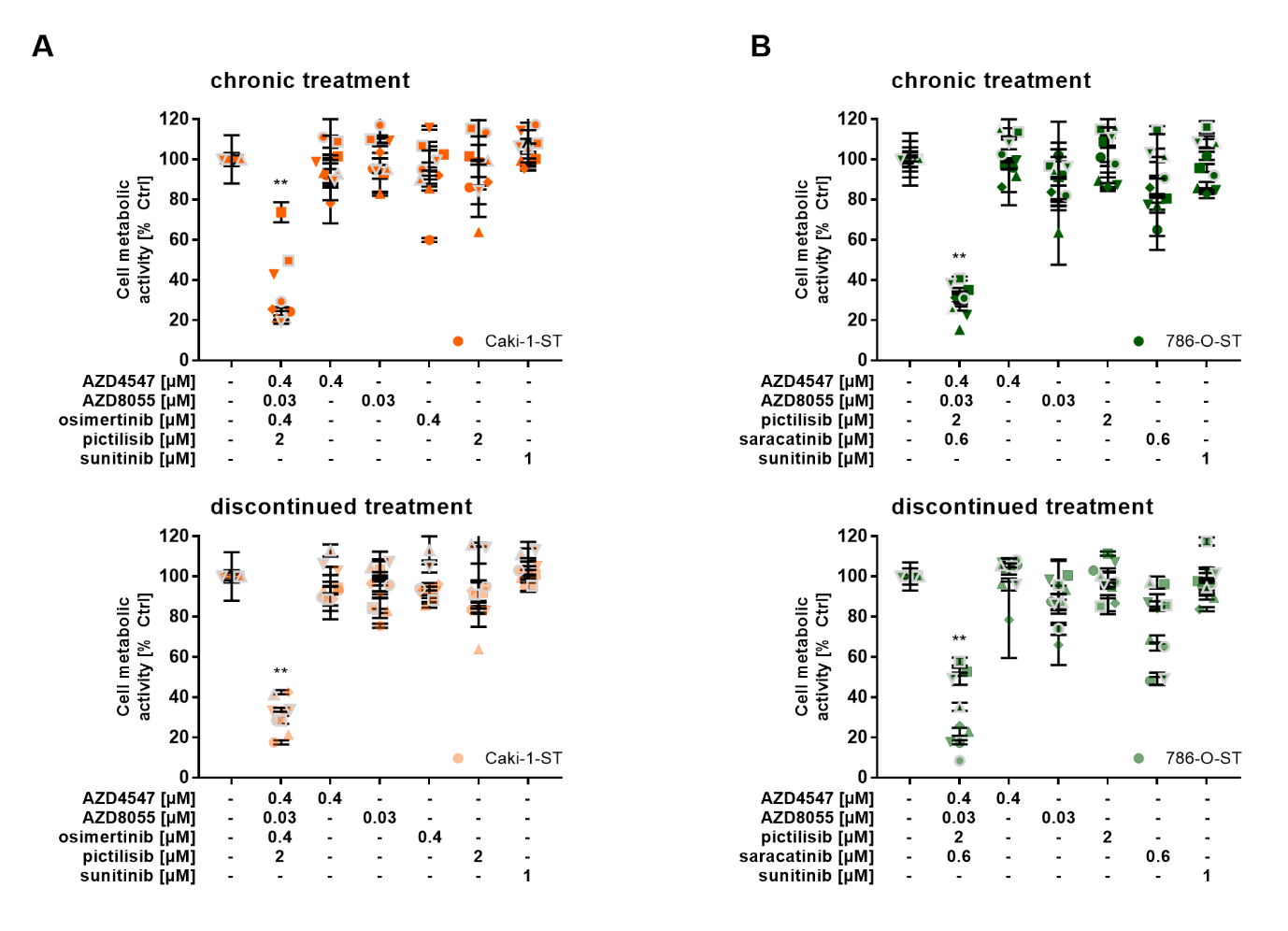
**

**Supplementary Figure S5: Maintenance of ODC activity following chronic or discontinued sunitinib treatment.**

Kinetics of insensitivity to sunitinib treatment in **A**) Caki-1-ST and **B**) 786-O-ST cells exposed to chronic treatment (left) with sunitinib and cells in which the sunitinib treatment was discontinued (right). Over this period the cells were treated once weekly and the cell metabolic activity readout was performed (N=3). Significances of **p < 0.005 define the difference of the ODC treatment to the Ctrl and each corresponding monotherapy determined with ordinary one- and two-way ANOVA with Tukey’s post hoc test. Error bars stand for the standard deviation.

**
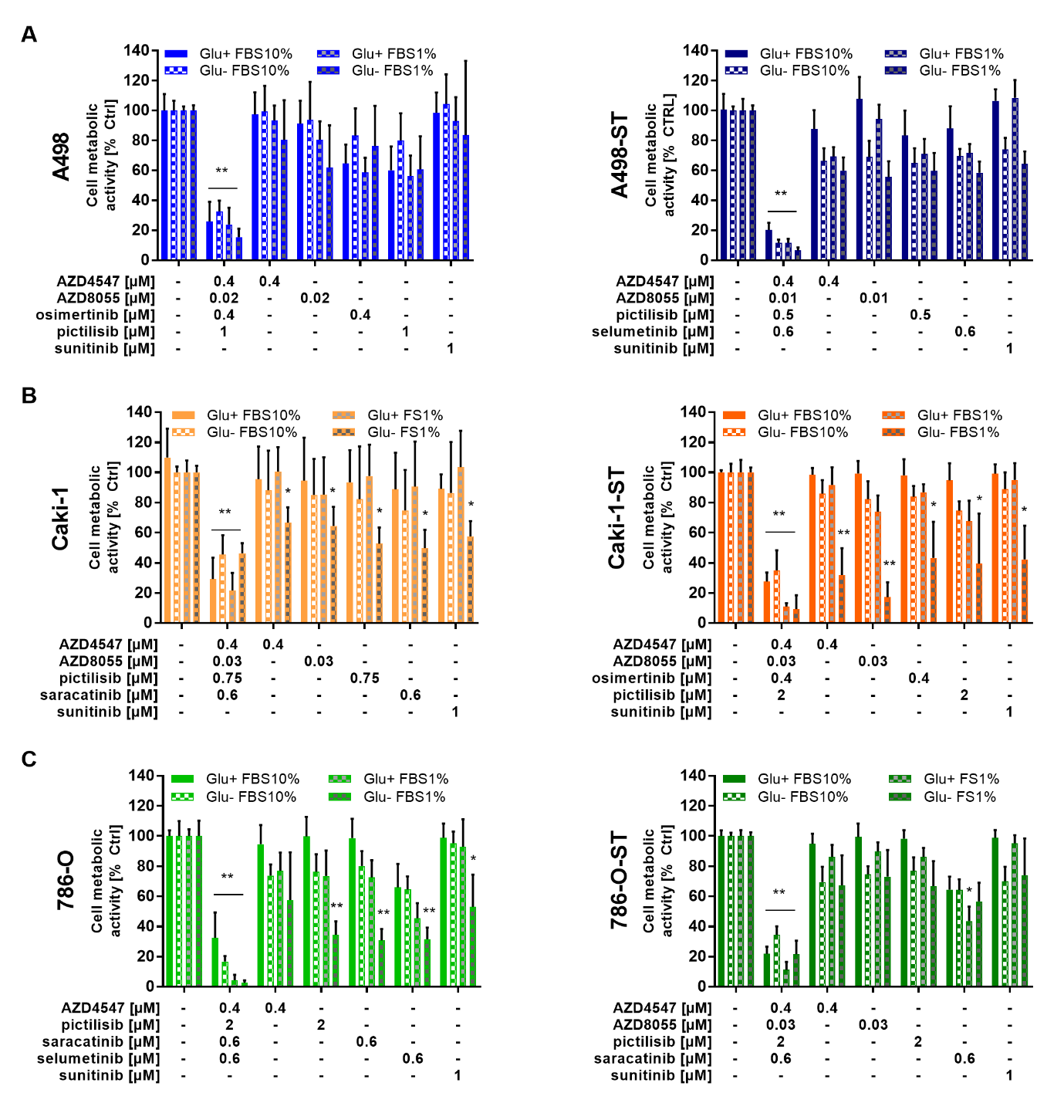
**

**Supplementary Figure S6: Efficacy of the ODC treatment as a function of the nutrient supply.**

Measurement of the efficacy of the Ctrl, ODC, corresponding monotherapies, and 1 µM sunitinib (positive Ctrl) in full, reduced or absence of FBS and glucose in **A**) A498 and A498-ST, **B**) Caki-1 and Caki-1-ST, as well as in **C**) 786-O and 786-O-ST cells (N =3). Significances of *p < 0.01 and **p < 0.005 define the difference of the ODC treatment to the Ctrl and each corresponding monotherapy determined with ordinary one- (in between the four conditions) and two-way ANOVA (Ctrl vs ODC vs all monotherapies) with Tukey’s post hoc test. Error bars stand for standard deviation.

**
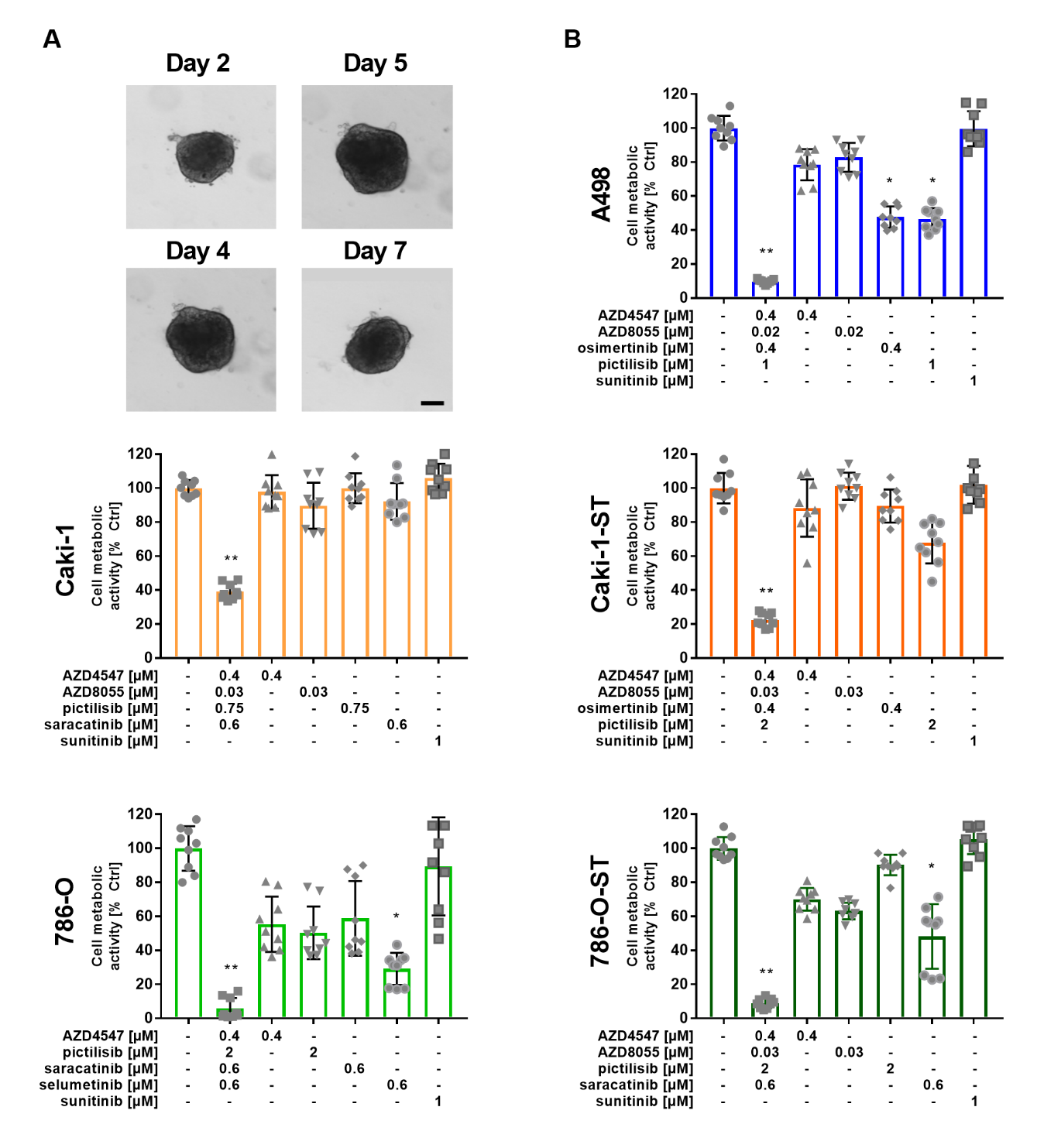
**

**Supplementary Figure S7: Efficacy of the ODC treatment in heterotypic 3D co-cultures.**

**A**) Representative pictures of A498 co-culture spheroids taken in accordance with both schedules applied in experimentation; (top) day 2-5 or (bottom) day 4-7. Scale bar represents 150 µm. **B**) Cell metabolic activity in the spheroids in all cell lines after treatment with Ctrl, ODC, corresponding monotherapies, and 1 µM sunitinib (positive control) following schedule day 2-5. Error bars represent the standard deviation (metabolic activity measurements, N = 2-3). Significances of *p < 0.01 and **p < 0.005 define the difference of the ODC treatment to the Ctrl and each corresponding monotherapies determined with ordinary one-way ANOVA with Tukey’s post hoc test. Error bars stand for standard deviation.

**
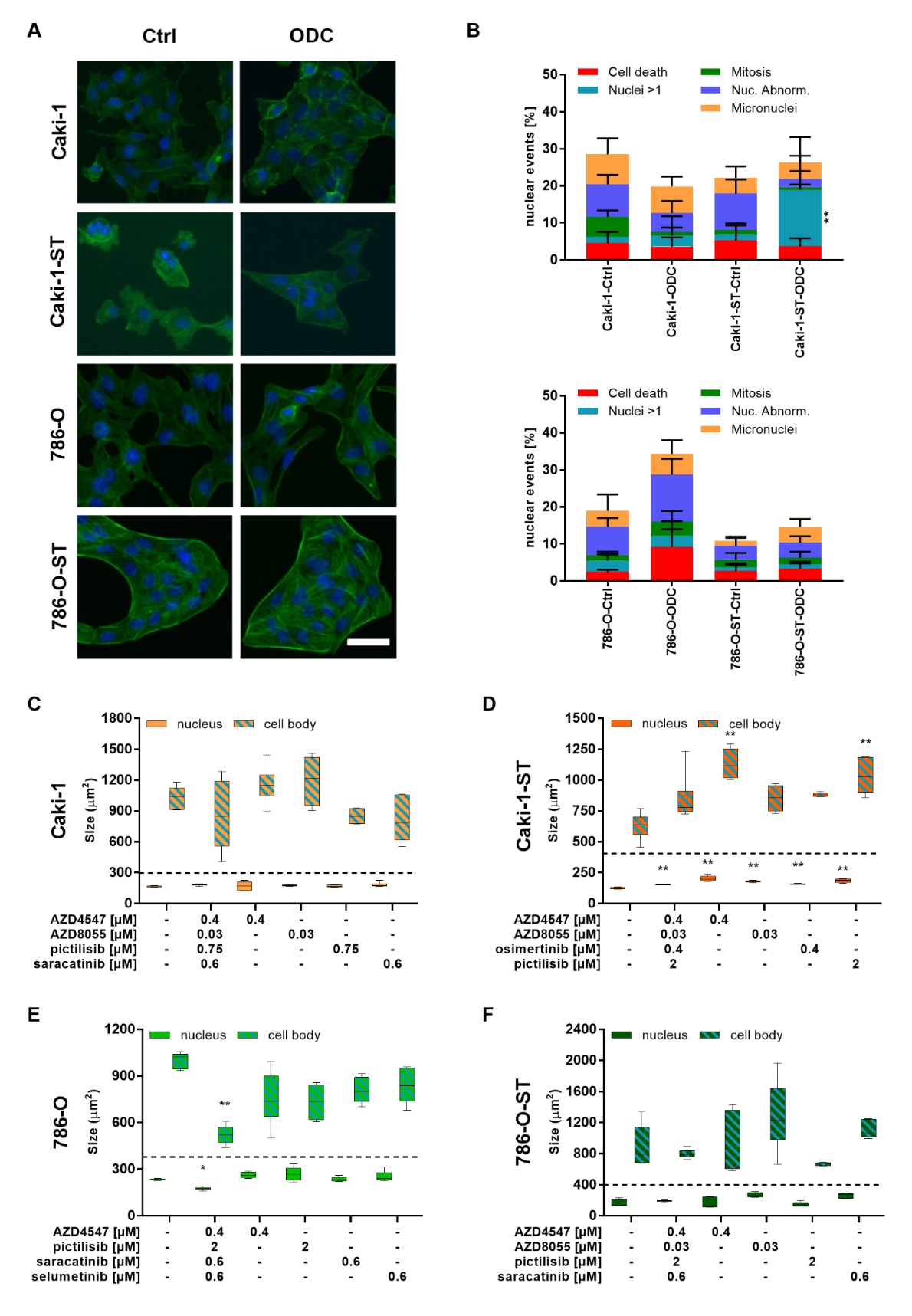
 Supplementary Figure S8: Induced abnormalities of cellular morphology after treatment.**

**A**) Representative images recorded after 24h of cell incubation with ODC or monotherapies. Caki-1(-ST) and 786-O(-ST) cells stained with phalloidin (f-actin, green) and Dapi (nucleus, blue). Scale bar represents 20 µm. B) Quantified nuclear abnormalities plotted for Ctrl and ODC of **B**) Caki-1(-ST) and 786-O(-ST) cells. The size of the cell body (striped, above dashed line) and nucleus (full, below dashed line) for **C**) Caki-1, **D**) Caki-1-ST, **E**) 786-O and **F**) 786-O-ST cells. Error bars represent the standard deviation (N = 2, 6 images of two separate experiments). Significances of two-tailed **p < 0.005 define the difference of the ODC treatment to the Ctrl determined with unpaired t-test. Error bars stand for standard deviation.


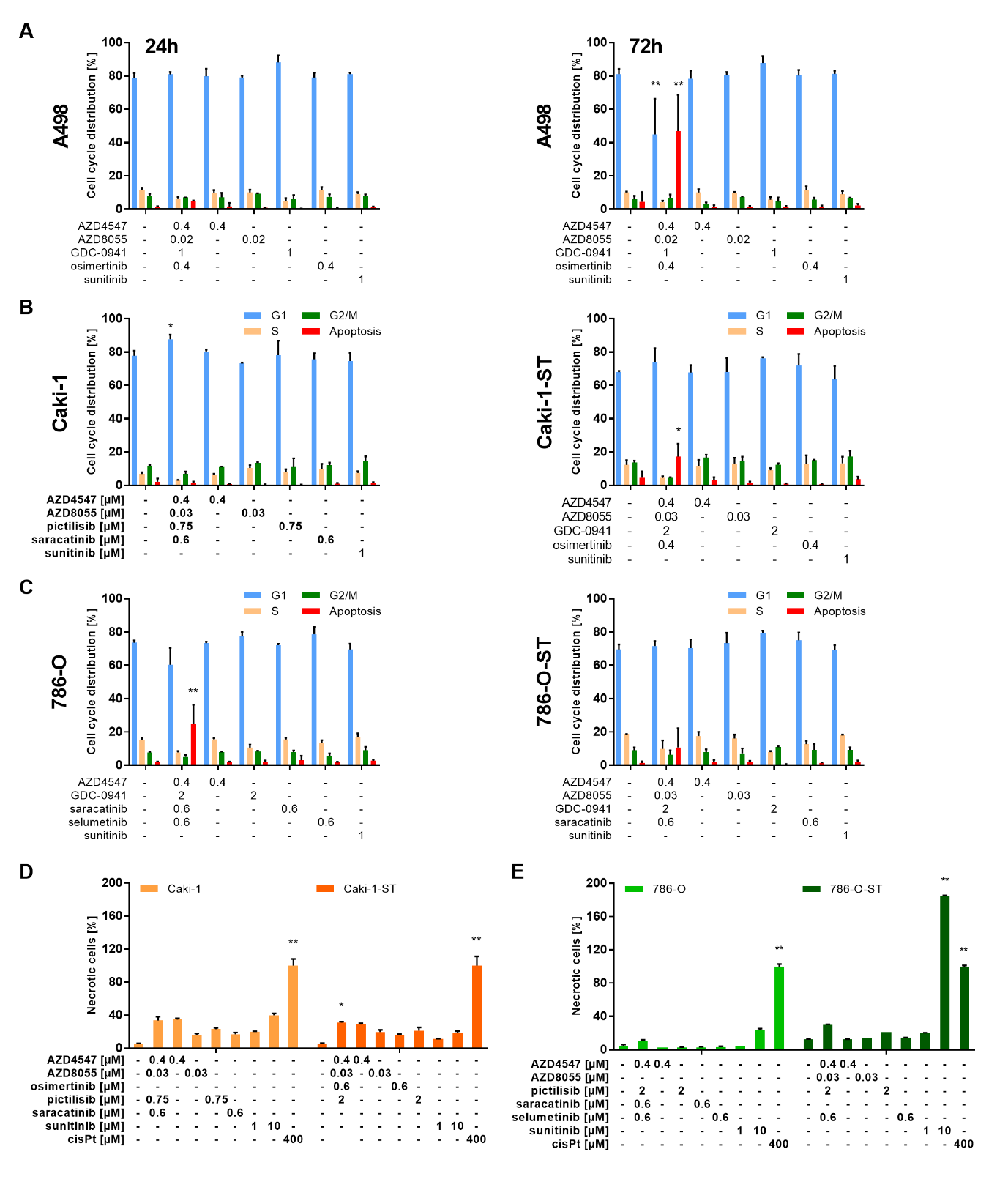


**Supplementary Figure S9: Treatment- and time-dependent alterations of the cell cycle and evaluation of necrotic events**

**A**) Cell cycle analysis of A498 cells after 24h (left graph) and 72h (right graph) presenting the cell cycle distribution within G1, S, G2/M phase and cell death. Cell cycle distribution after 72h treatment of **B**) Caki-1 (left) and Caki-1-ST cells (right) as well as of **C**) 786-O (left) and 786-O-ST cells (right). Induction of necrosis **D**) in Caki-1 and Caki-1-ST as well as in **E**) 786-O and 786-O-ST cells after 72h treatment with ODC, corresponding monotherapies and positive controls (1 µM sunitinib, 10 µM sunitinib and 400 µM cisplatin). Error bars represent the standard deviation (N = 3). Significances of *p < 0.01 and **p < 0.005 define the difference of the ODC treatment to the Ctrl determined with unpaired t-test. Error bars stand for standard deviation.

**
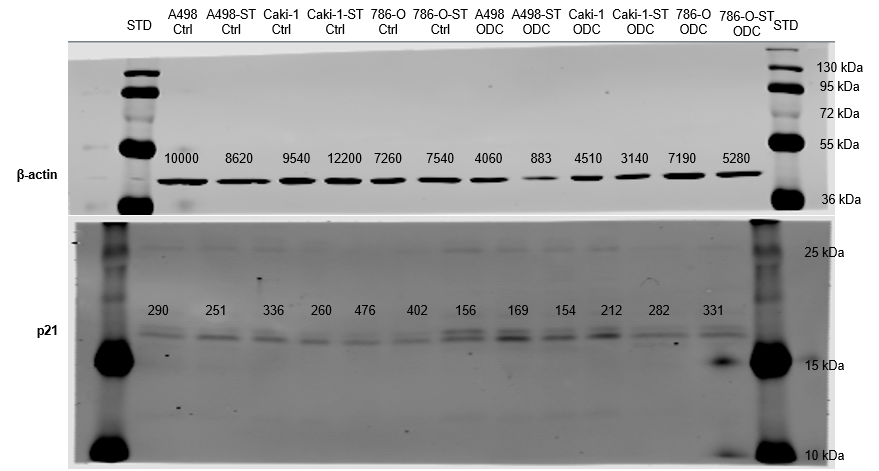
**

**
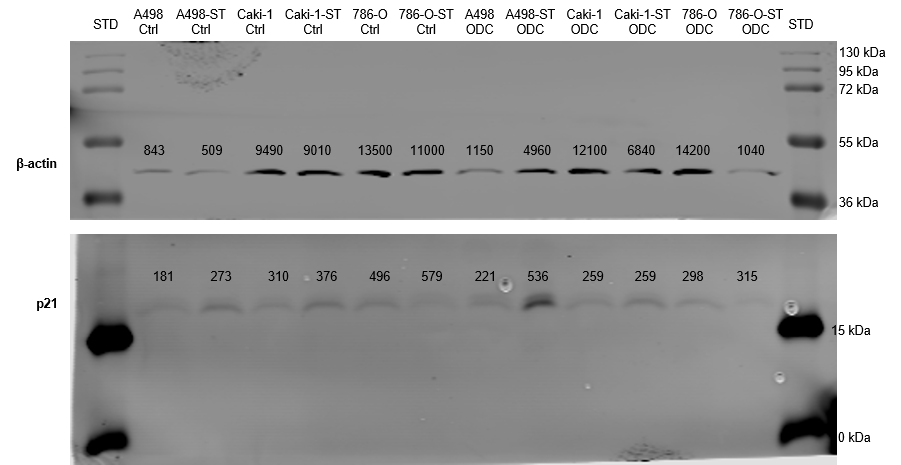

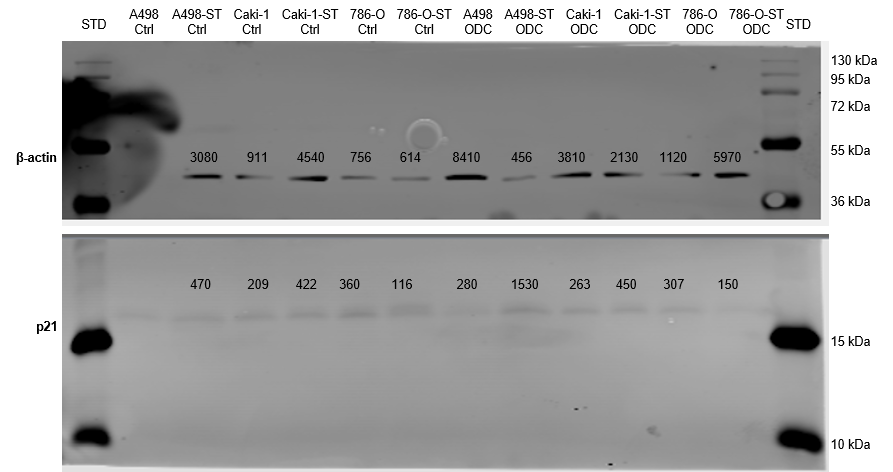

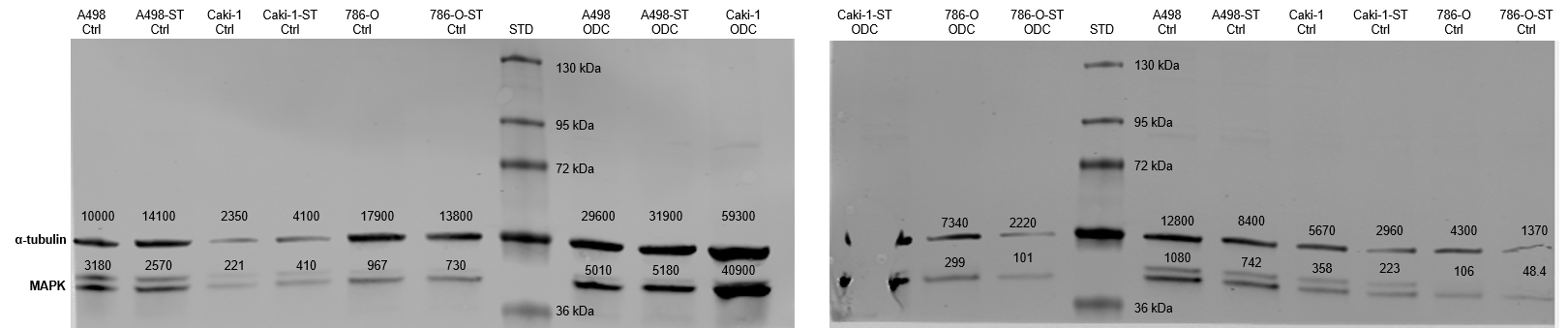

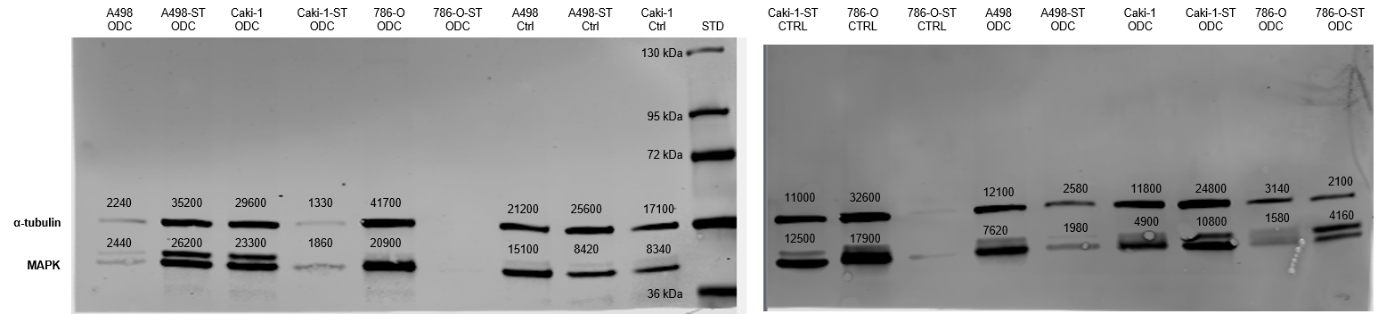
**

**Supplementary Figure S10: Full length unprocessed western blot results of MAPK and p21 in RCC cell lines after 2h of treatment.**

Western blot analysis of MAPK, p21 and housekeeping genes β-actin and α-tubulin in naïve and ST-RCC cell lines after 2h exposure to Ctrl or cell type specific ODC treatment. Protein levels of β-actin (45 kDa) and p21 (21 kDa) as well as α-tubulin (50 kDa) and MAPK (42 kDa) were quantified. Each sample was loaded at a concentration of 30 µg/µL. Images were taken with a Licor Odyssey CLx scanner at default exposure setting.

**
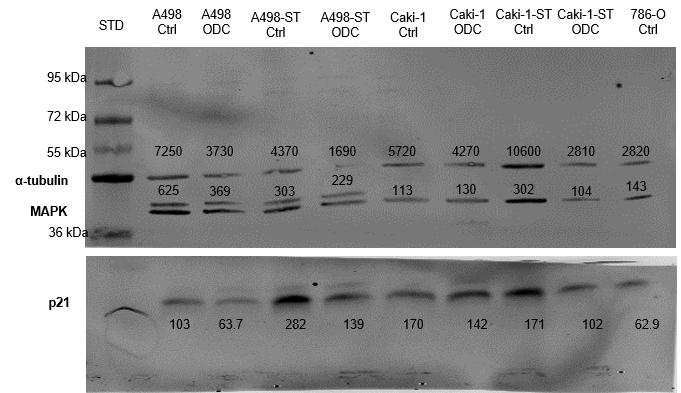
**

**
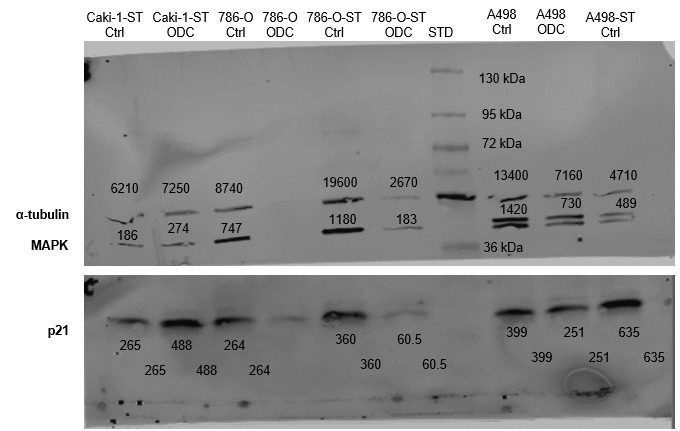
**

**
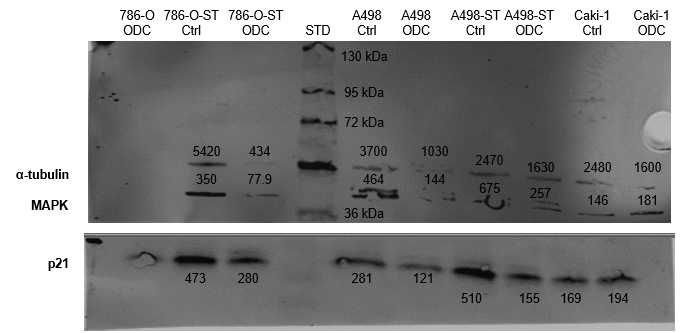
**

**Supplementary Figure S11: Full length unprocessed western blot results of MAPK and p21 in RCC cell lines after 24h of treatment.**

Western blot analysis of MAPK, p21 and housekeeping gene α-tubulin in naïve and ST-RCC cell lines after 24h exposure to Ctrl or cell type specific ODC treatment. Each sample was loaded at a concentration of 30 µg/µL. Images were taken with the Licor Odyssey CLx scanner at default exposure setting.

**Supplementary Table S1.** Selected drugs and their targets

| **Compound** | **Cellular target** | **Original indication** | **Development** |
| --- | --- | --- | --- |
| **alisertib**  **(MLN8237)*** | Aurora kinase A, B | Pediatric cancers(1)  Solid malignancies(2, 3)  T cell lymphoma(4) | Phase I-III |
| **axitinib** | VEGFR1/2/3, PDGFRβ, c-Kit | Advanced renal cell carcinoma(5) | Approved |
| **AZD4547** | FGFR1/2/3, FGFR4, VEGFR2 | Breast cancer  ***(NCT01202591)***  **(adeno-)carcinoma of the esophagus, gastroesophageal junction or stomach**  ***(****Eudract:* *2011-000642-37)* | Phase IIa |
| **AZD8055** | mTORC1/2 | Advanced solid tumors and lymphoma(6) | Phase I/II |
| **crenolanib**  **(CP-868596)** | PDGFRα/β, FLT3, c-Kit, VEGFR-2, TIE-2, FGFR-2, EGFR, erbB2, Src | Acute myeloid leukemia(7) | Phase III |
| **icaritin** | JAK/STAT3 | Advanced hepatocellular carcinoma *(NCT03236649, NCT03236636)* | Phase III |
| **osimertinib** | EGFR | Non-small cell lung cancer(8) | Approved |
| **pictilisib**  **(GDC-0941)** | PI3Kα/δ | Advanced or metastatic  breast cancer(9) | Phase II |
| **saracatinib**  **(AZD0530)*** | Src, Bcr-Abl, Lck | Metastatic melanoma(10)  Metastatic head and neck squamous cell carcinoma(11)  T-cell acute lymphoblastic leukemia(12) | Phase II |
| **selumetinib** | MEK1/2, ERK1/2 | Metastatic uveal melanoma(13) | Phase III |
| **alisertib**  **(MLN8237)*** | Aurora kinase A, B | Pediatric cancers(1)  Solid malignancies(2, 3)  T cell lymphoma(4) | Phase I-III |

Drugs are listed in alphabetical order and cellular targets for each drug are given in decreasing selectivity order. *abandoned in cancer treatment; used for Alzheimer; recently tested in T-ALL and AML treatment.

**Supplementary Table S2.** Characteristics of the cell lines used

|  | **cancerous** | | | **non-cancerous** | | |
| --- | --- | --- | --- | --- | --- | --- |
| **Cells** | **A498** | **Caki-1** | **786-O** | **ECRF24** | **HEK-293T** | **NHDFα** |
| **Origin** | RCC primary tumor | ccRCC metastasis | RCC primary tumor | vascular endothelium | embryonic kidney | juvenile foreskin |
| **Morphology** | epithelial | epithelial | epithelial | endothelial | epithelial | fibroblast |
| **VHL status** | mutant | wt | mutant | N/A | wt | N/A |
| **p53 status** | wt | wt | mutant | N/A | wt | N/A |
| **PTEN** | wt | wt | mutant | N/A | wt | N/A |
| **Sensitivity to 10 µM sunitinib** | 70%(14) | 80%(14) | 70%(15) | N/A | N/A | N/A |

wt = wild type, N/A = not applicable

**Supplementary Table S3.** Cross-validation of all RCC-specific ODCs

|  | **Efficacy (% Ctrl)** | | | | | | |
| --- | --- | --- | --- | --- | --- | --- | --- |
| **Cells** | **A498** | **A498-ST** | **Caki-1** | **Caki-1-ST** | **786-O** | **786-O-ST** | **ECRF24** |
| **A498** | **43.85** | 50.05 | 38.18 | 34.70 | 39.74 | 45.03 | 57.66 |
| ±SD | 4.2 | 4.9 | 2.1 | 3.8 | 16.5 | 2.1 | 16.7 |
| **A498-ST** | 36.12 | **32.78** | 29.19 | 23.66 | 17.64 | 39.49 | 65.94 |
| ±SD | 5.8 | 6.2 | 4.3 | 4.7 | 8.1 | 13.0 | 10.9 |
| **Caki-1** | 28.33 | 36.46 | **28.50** | 28.13 | 25.39 | 28.44 | 32.41 |
| ±SD | 3.8 | 5.1 | 3.9 | 4.4 | 1.7 | 6.6 | 5.6 |
| **Caki-1-ST** | 33.42 | 33.42 | 40.09 | **31.01** | 29.07 | 22.15 | 30.69 |
| ±SD | 7.1 | 8.4 | 7.9 | 4.7 | 4.3 | 12.5 | 6.0 |
| **786-O** | 40.34 | 58.26 | 30.91 | 29.07 | **13.51** | 26.34 | 44.69 |
| ±SD | 7.6 | 7.2 | 7.5 | 7.5 | 2.0 | 13.8 | 3.2 |
| **786-O-ST** | 58.08 | 65.55 | 47.35 | 53.63 | 50.48 | **42.91** | 76.46 |
| ±SD | 5.5 | 6.1 | 4.3 | 6.1 | 5.0 | 1.5 | 8.7 |
| **ECRF24** | 47.90 | 39.11 | 44.93 | 39.66 | 43.61 | 39.19 | **46.48** |
| ±SD | 16.1 | 26.8 | 16.1 | 15.6 | 16.7 | 13.3 | 20.2 |
| **HEK-293T** | 83.15 | 79.23 | 40.43 | 65.12 | 86.29 | 35.76 | 110.25 |
| ±SD | 3.2 | 4.5 | 5.9 | 6.4 | 4.6 | 3.4 | 6.8 |

**Supplementary Videos S1-S2**

**3D co-culture spheroid formation containing A498-ST cells**

Representative movies (**S1**, bright field and **S2,** fluorescence stained) recorded for 24 hours of an A498-ST 3D co-culture capturing the kinetic formation of a 3D spheroid. 3D co-cultures harbor 70% tumor cells (A498-ST, green), 20% fibroblasts (NHDFα, blue) and 10% endothelial cells (ECRF24, red). Scalebar represents 1000 µm.

**References**

1. Niu H, Manfredi M, Ecsedy JA. Scientific Rationale Supporting the Clinical Development Strategy for the Investigational Aurora A Kinase Inhibitor Alisertib in Cancer. Front Oncol. 2015;5:189.

2. Dees EC, Cohen RB, von Mehren M, Stinchcombe TE, Liu H, Venkatakrishnan K, et al. Phase I study of aurora A kinase inhibitor MLN8237 in advanced solid tumors: safety, pharmacokinetics, pharmacodynamics, and bioavailability of two oral formulations. Clin Cancer Res. 2012;18(17):4775-84.

3. Cervantes A, Elez E, Roda D, Ecsedy J, Macarulla T, Venkatakrishnan K, et al. Phase I pharmacokinetic/pharmacodynamic study of MLN8237, an investigational, oral, selective aurora a kinase inhibitor, in patients with advanced solid tumors. Clin Cancer Res. 2012;18(17):4764-74.

4. Liewer S, Huddleston A. Alisertib: a review of pharmacokinetics, efficacy and toxicity in patients with hematologic malignancies and solid tumors. Expert Opin Investig Drugs. 2018;27(1):105-12.

5. Tzogani K, Skibeli V, Westgaard I, Dalhus M, Thoresen H, Slot KB, et al. The European Medicines Agency approval of axitinib (Inlyta) for the treatment of advanced renal cell carcinoma after failure of prior treatment with sunitinib or a cytokine: summary of the scientific assessment of the committee for medicinal products for human use. Oncologist. 2015;20(2):196-201.

6. Naing A, Aghajanian C, Raymond E, Olmos D, Schwartz G, Oelmann E, et al. Safety, tolerability, pharmacokinetics and pharmacodynamics of AZD8055 in advanced solid tumours and lymphoma. Br J Cancer. 2012;107(7):1093-9.

7. Sutamtewagul G, Vigil CE. Clinical use of FLT3 inhibitors in acute myeloid leukemia. Onco Targets Ther. 2018;11:7041-52.

8. Soria JC, Ohe Y, Vansteenkiste J, Reungwetwattana T, Chewaskulyong B, Lee KH, et al. Osimertinib in Untreated EGFR-Mutated Advanced Non-Small-Cell Lung Cancer. N Engl J Med. 2018;378(2):113-25.

9. Krop I, Johnston S, Mayer IA, Dickler M, Ganju V, Forero-Torres A, et al. Abstract S2-02: The FERGI phase II study of the PI3K inhibitor pictilisib (GDC-0941) plus fulvestrant vs fulvestrant plus placebo in patients with ER+, aromatase inhibitor (AI)-resistant advanced or metastatic breast cancer – Part I results. Cancer Research. 2015;75(9 Supplement):S2-02.

10. Gangadhar TC, Clark JI, Karrison T, Gajewski TF. Phase II study of the Src kinase inhibitor saracatinib (AZD0530) in metastatic melanoma. Invest New Drugs. 2013;31(3):769-73.

11. Fury MG, Baxi S, Shen R, Kelly KW, Lipson BL, Carlson D, et al. Phase II study of saracatinib (AZD0530) for patients with recurrent or metastatic head and neck squamous cell carcinoma (HNSCC). Anticancer Res. 2011;31(1):249-53.

12. Buffiere A, Accogli T, Saint-Paul L, Lucchi G, Uzan B, Ballerini P, et al. Saracatinib impairs maintenance of human T-ALL by targeting the LCK tyrosine kinase in cells displaying high level of lipid rafts. Leukemia. 2018;32(9):2062-5.

13. Carvajal RD, Piperno-Neumann S, Kapiteijn E, Chapman PB, Frank S, Joshua AM, et al. Selumetinib in Combination With Dacarbazine in Patients With Metastatic Uveal Melanoma: A Phase III, Multicenter, Randomized Trial (SUMIT). J Clin Oncol. 2018;36(12):1232-9.

14. Mahalingam D, Espitia CM, Medina EC, Esquivel JA, 2nd, Kelly KR, Bearss D, et al. Targeting PIM kinase enhances the activity of sunitinib in renal cell carcinoma. Br J Cancer. 2011;105(10):1563-73.

15. Xin H, Zhang C, Herrmann A, Du Y, Figlin R, Yu H. Sunitinib inhibition of Stat3 induces renal cell carcinoma tumor cell apoptosis and reduces immunosuppressive cells. Cancer Res. 2009;69(6):2506-13.
