# Supplementary material for: Identification of low-dose multidrug combinations for sunitinib-naive and pre-treated renal cell carcinoma
Source: Br J Cancer. 2020 May 22;123(4):556–67. doi: 10.1038/s41416-020-0890-y (PMC7435198; doi:10.1038/s41416-020-0890-y)
Supplement: Supplementary file 2 — Supplementary Tables [file 41416_2020_890_MOESM2_ESM.docx]

**Rausch *et al*. Tables**

**Identification of low-dose multidrug combinations for sunitinib naive and pre-treated renal cell carcinoma**

**Magdalena Rausch^1,2^, Andrea Weiss^1^, Joanna Achkhanian^1^, Andrei Rotari^1^, Patrycja Nowak-Sliwinska^1,2,^***

^1^Molecular Pharmacology Group, Institute of Pharmaceutical Sciences of Western Switzerland,1 Rue Michel-Servet, 1211, Geneva 4, Switzerland;

^2^Translational Research Center in Oncohaematology, 1 Rue Michel-Servet, 1211, Geneva 4, Switzerland

**Main Tables**

**Supplementary Tables S1-S3**

**Table 1. Cell line-specific four-drug low-dose combinations and their efficacy in cell metabolic activity inhibition identified in the TGMO based search.**

| **Cells** | | **A498** | **A498-ST** | **Caki-1** | **Caki-1-ST** | **786-O** | **786-O-ST** | **ECRF24** |
| --- | --- | --- | --- | --- | --- | --- | --- | --- |
| **Drugs** | **MPC [µM]** | **Dose in ODC [µM]** | | | | | | |
| axitinib | 0.02 | - | - | - | - | - | - | 0.02 |
| AZD4547 | 0.4 | 0.4 | 0.4 | 0.4 | 0.4 | 0.4 | 0.4 | - |
| AZD8055 | 0.03 | 0.02 | 0.01 | 0.03 | 0.03 | - | 0.03 | - |
| osimertinib | 0.4 | 0.4 | - | - | 0.4 | - | - | - |
| pictilisib | 2.0 | 1 | 0.5 | 0.75 | 2.0 | 2.0 | 2.0 | 0.5 |
| saracatinib | 0.6 | - | - | 0.6 | - | 0.6 | 0.6 | 0.6 |
| selumetinib | 0.6 | - | 0.6 | - | - | 0.6 | - | 0.6 |
| **Efficacy (% Ctrl)**  ±SD | | **66.3**  11 | **71.6**  8 | **70.0**  8 | **69.6**  7 | **67.8**  16 | **70.3**  9 | **53.5**  5 |

**Supplementary Table S1.** Selected drugs and their targets

| **Compound** | **Cellular target** | **Original indication** | **Development** |
| --- | --- | --- | --- |
| **alisertib**  **(MLN8237)*** | Aurora kinase A, B | Pediatric cancers(1)  Solid malignancies(2, 3)  T cell lymphoma(4) | Phase I-III |
| **axitinib** | VEGFR1/2/3, PDGFRβ, c-Kit | Advanced renal cell carcinoma(5) | Approved |
| **AZD4547** | FGFR1/2/3, FGFR4, VEGFR2 | Breast cancer  ***(NCT01202591)***  **(adeno-)carcinoma of the esophagus, gastroesophageal junction or stomach**  ***(****Eudract:* *2011-000642-37)* | Phase IIa |
| **AZD8055** | mTORC1/2 | Advanced solid tumors and lymphoma(6) | Phase I/II |
| **crenolanib**  **(CP-868596)** | PDGFRα/β, FLT3, c-Kit, VEGFR-2, TIE-2, FGFR-2, EGFR, erbB2, Src | Acute myeloid leukemia(7) | Phase III |
| **icaritin** | JAK/STAT3 | Advanced hepatocellular carcinoma *(NCT03236649, NCT03236636)* | Phase III |
| **osimertinib** | EGFR | Non-small cell lung cancer(8) | Approved |
| **pictilisib**  **(GDC-0941)** | PI3Kα/δ | Advanced or metastatic  breast cancer(9) | Phase II |
| **saracatinib**  **(AZD0530)*** | Src, Bcr-Abl, Lck | Metastatic melanoma(10)  Metastatic head and neck squamous cell carcinoma(11)  T-cell acute lymphoblastic leukemia(12) | Phase II |
| **selumetinib** | MEK1/2, ERK1/2 | Metastatic uveal melanoma(13) | Phase III |
| **alisertib**  **(MLN8237)*** | Aurora kinase A, B | Pediatric cancers(1)  Solid malignancies(2, 3)  T cell lymphoma(4) | Phase I-III |

Drugs are listed in alphabetical order and cellular targets for each drug are given in decreasing selectivity order. *abandoned in cancer treatment; used for Alzheimer; recently tested in T-ALL and AML treatment.

**Supplementary Table S2.** Characteristics of the cell lines used

|  | **cancerous** | | | **non-cancerous** | | |
| --- | --- | --- | --- | --- | --- | --- |
| **Cells** | **A498** | **Caki-1** | **786-O** | **ECRF24** | **HEK-293T** | **NHDFα** |
| **Origin** | RCC primary tumor | ccRCC metastasis | RCC primary tumor | vascular endothelium | embryonic kidney | juvenile foreskin |
| **Morphology** | epithelial | epithelial | epithelial | endothelial | epithelial | fibroblast |
| **VHL status** | mutant | wt | mutant | N/A | wt | N/A |
| **p53 status** | wt | wt | mutant | N/A | wt | N/A |
| **PTEN** | wt | wt | mutant | N/A | wt | N/A |
| **Sensitivity to 10 µM sunitinib** | 70%(14) | 80%(14) | 70%(15) | N/A | N/A | N/A |

wt = wild type, N/A = not applicable

**Supplementary Table S3.** Cross-validation of all RCC-specific ODCs

|  | **Efficacy (% Ctrl)** | | | | | | |
| --- | --- | --- | --- | --- | --- | --- | --- |
| **Cells** | **A498** | **A498-ST** | **Caki-1** | **Caki-1-ST** | **786-O** | **786-O-ST** | **ECRF24** |
| **A498** | **43.85** | 50.05 | 38.18 | 34.70 | 39.74 | 45.03 | 57.66 |
| ±SD | 4.2 | 4.9 | 2.1 | 3.8 | 16.5 | 2.1 | 16.7 |
| **A498-ST** | 36.12 | **32.78** | 29.19 | 23.66 | 17.64 | 39.49 | 65.94 |
| ±SD | 5.8 | 6.2 | 4.3 | 4.7 | 8.1 | 13.0 | 10.9 |
| **Caki-1** | 28.33 | 36.46 | **28.50** | 28.13 | 25.39 | 28.44 | 32.41 |
| ±SD | 3.8 | 5.1 | 3.9 | 4.4 | 1.7 | 6.6 | 5.6 |
| **Caki-1-ST** | 33.42 | 33.42 | 40.09 | **31.01** | 29.07 | 22.15 | 30.69 |
| ±SD | 7.1 | 8.4 | 7.9 | 4.7 | 4.3 | 12.5 | 6.0 |
| **786-O** | 40.34 | 58.26 | 30.91 | 29.07 | **13.51** | 26.34 | 44.69 |
| ±SD | 7.6 | 7.2 | 7.5 | 7.5 | 2.0 | 13.8 | 3.2 |
| **786-O-ST** | 58.08 | 65.55 | 47.35 | 53.63 | 50.48 | **42.91** | 76.46 |
| ±SD | 5.5 | 6.1 | 4.3 | 6.1 | 5.0 | 1.5 | 8.7 |
| **ECRF24** | 47.90 | 39.11 | 44.93 | 39.66 | 43.61 | 39.19 | **46.48** |
| ±SD | 16.1 | 26.8 | 16.1 | 15.6 | 16.7 | 13.3 | 20.2 |
| **HEK-293T** | 83.15 | 79.23 | 40.43 | 65.12 | 86.29 | 35.76 | 110.25 |
| ±SD | 3.2 | 4.5 | 5.9 | 6.4 | 4.6 | 3.4 | 6.8 |

**References**

1. Niu H, Manfredi M, Ecsedy JA. Scientific Rationale Supporting the Clinical Development Strategy for the Investigational Aurora A Kinase Inhibitor Alisertib in Cancer. Front Oncol. 2015;5:189.

2. Dees EC, Cohen RB, von Mehren M, Stinchcombe TE, Liu H, Venkatakrishnan K, et al. Phase I study of aurora A kinase inhibitor MLN8237 in advanced solid tumors: safety, pharmacokinetics, pharmacodynamics, and bioavailability of two oral formulations. Clin Cancer Res. 2012;18(17):4775-84.

3. Cervantes A, Elez E, Roda D, Ecsedy J, Macarulla T, Venkatakrishnan K, et al. Phase I pharmacokinetic/pharmacodynamic study of MLN8237, an investigational, oral, selective aurora a kinase inhibitor, in patients with advanced solid tumors. Clin Cancer Res. 2012;18(17):4764-74.

4. Liewer S, Huddleston A. Alisertib: a review of pharmacokinetics, efficacy and toxicity in patients with hematologic malignancies and solid tumors. Expert Opin Investig Drugs. 2018;27(1):105-12.

5. Tzogani K, Skibeli V, Westgaard I, Dalhus M, Thoresen H, Slot KB, et al. The European Medicines Agency approval of axitinib (Inlyta) for the treatment of advanced renal cell carcinoma after failure of prior treatment with sunitinib or a cytokine: summary of the scientific assessment of the committee for medicinal products for human use. Oncologist. 2015;20(2):196-201.

6. Naing A, Aghajanian C, Raymond E, Olmos D, Schwartz G, Oelmann E, et al. Safety, tolerability, pharmacokinetics and pharmacodynamics of AZD8055 in advanced solid tumours and lymphoma. Br J Cancer. 2012;107(7):1093-9.

7. Sutamtewagul G, Vigil CE. Clinical use of FLT3 inhibitors in acute myeloid leukemia. Onco Targets Ther. 2018;11:7041-52.

8. Soria JC, Ohe Y, Vansteenkiste J, Reungwetwattana T, Chewaskulyong B, Lee KH, et al. Osimertinib in Untreated EGFR-Mutated Advanced Non-Small-Cell Lung Cancer. N Engl J Med. 2018;378(2):113-25.

9. Krop I, Johnston S, Mayer IA, Dickler M, Ganju V, Forero-Torres A, et al. Abstract S2-02: The FERGI phase II study of the PI3K inhibitor pictilisib (GDC-0941) plus fulvestrant vs fulvestrant plus placebo in patients with ER+, aromatase inhibitor (AI)-resistant advanced or metastatic breast cancer – Part I results. Cancer Research. 2015;75(9 Supplement):S2-02.

10. Gangadhar TC, Clark JI, Karrison T, Gajewski TF. Phase II study of the Src kinase inhibitor saracatinib (AZD0530) in metastatic melanoma. Invest New Drugs. 2013;31(3):769-73.

11. Fury MG, Baxi S, Shen R, Kelly KW, Lipson BL, Carlson D, et al. Phase II study of saracatinib (AZD0530) for patients with recurrent or metastatic head and neck squamous cell carcinoma (HNSCC). Anticancer Res. 2011;31(1):249-53.

12. Buffiere A, Accogli T, Saint-Paul L, Lucchi G, Uzan B, Ballerini P, et al. Saracatinib impairs maintenance of human T-ALL by targeting the LCK tyrosine kinase in cells displaying high level of lipid rafts. Leukemia. 2018;32(9):2062-5.

13. Carvajal RD, Piperno-Neumann S, Kapiteijn E, Chapman PB, Frank S, Joshua AM, et al. Selumetinib in Combination With Dacarbazine in Patients With Metastatic Uveal Melanoma: A Phase III, Multicenter, Randomized Trial (SUMIT). J Clin Oncol. 2018;36(12):1232-9.

14. Mahalingam D, Espitia CM, Medina EC, Esquivel JA, 2nd, Kelly KR, Bearss D, et al. Targeting PIM kinase enhances the activity of sunitinib in renal cell carcinoma. Br J Cancer. 2011;105(10):1563-73.

15. Xin H, Zhang C, Herrmann A, Du Y, Figlin R, Yu H. Sunitinib inhibition of Stat3 induces renal cell carcinoma tumor cell apoptosis and reduces immunosuppressive cells. Cancer Res. 2009;69(6):2506-13.
